# Mortality Risk Following a Household Suicide

**DOI:** 10.1001/jamanetworkopen.2025.45286

**Published:** 2025-11-25

**Authors:** Flávia Alves, Elisângela da Silva Rodrigues, Lidiane Toledo, Júlia M. Pescarini, Rodrigo Lins Rodrigues, John A. Naslund, Maurício L. Barreto, Vikram Patel, Daiane B. Machado

**Affiliations:** 1 Center of Data and Knowledge Integration for Health, Instituto Gonçalo Moniz, Fundação Oswaldo Cruz, Salvador, Bahia, Brazil; 2 Department of Global Health and Social Medicine, Harvard Medical School, Boston, Massachusetts; 3 Federal University of Ceará, Campus Jardins de Anita, Itapajé, Ceará, Brazil; 4 Department of Infectious Disease Epidemiology, and Epidemiology and Population Health, Faculty of Epidemiology and Population Health, London School of Hygiene and Tropical Medicine, London, United Kingdom; 5 Federal Rural University of Pernambuco, Dois irmãos Campus, Recife, Pernambuco, Brazil

## Abstract

**Importance:**

The broader mortality risks faced by household members following a suicide remain poorly understood, particularly in low- and middle-income countries.

**Objectives:**

To estimate the risk of all-cause and cause-specific mortality among surviving household members after a suicide within the household and to identify individual and contextual factors associated with mortality risk using data from a national population-based cohort in Brazil.

**Design, setting, and participants:**

This nationwide cohort study used data from the 100 Million Brazilian Cohort linked to the Mortality Information System (SIM) (2001-2018). All individuals who lived in a household with a suicide index case were considered exposed. Data were analyzed from March 2023 to September 2025.

**Main Outcomes and Measures:**

All-cause mortality, cause-specific mortality, and suicide. Outcomes were derived from the national mortality tracking system. The adjusted hazard ratio of all-cause and specific-cause mortality was calculated using a multivariate, time-varying Cox regression. The risk factors associated with increased mortality, including characteristics of the index case, surviving household members, household conditions, and the timing since the suicide event were also analyzed.

**Results:**

The cohort included 101 million individuals, of which there were 47 982 suicide index cases identified. Household members exposed to a suicide (11 070 [7%] Black, 82 407 [53%] Parda, and 57 726 [37%] White) had a 32% higher risk of all-cause mortality (adjusted hazard ratio [aHR], 1.32; 95% CI, 1.28-1.36). The risk of suicide among exposed individuals was more than 4 times higher (aHR, 4.42; 95% CI, 3.86-5.07), with 101 of these deaths (44%) occurring within 2 years of the index suicide case. The population attributable fraction for suicide was 77%. Elevated risks were also observed for other external causes (eg, assault and falls) and nonexternal causes (eg, neoplasms and circulatory and respiratory diseases). Mortality risk was highest when the index case was female and younger, among male survivors, and individuals aged 25 to 59 years. Better household conditions were associated with lower risks of both suicide and all-cause mortality.

**Conclusions and Relevance:**

Exposure to suicide within the household was associated with a substantial increase in both suicide-specific and all-cause mortality among surviving household members, particularly in the immediate aftermath. These findings underscore the urgent need to incorporate targeted postvention strategies into comprehensive suicide prevention efforts.

## Key Points

**Question:**

Does household exposure to suicide increase mortality among surviving members, and what risk factors and temporal patterns are involved?

**Findings:**

In this cohort study of over 100 million Brazilians, household suicide exposure was associated with a 32% higher risk of all-cause mortality and a 4-fold higher risk of suicide, with over half of suicides occurred within 2 years following the index case. Risks were greatest when the index cases were young or female and among those in poor housing conditions.

**Meaning:**

These findings suggest that household suicide exposure confers substantial time-sensitive mortality risks, underscoring the need for targeted early postvention strategies.

## Teaser

This cohort study evaluates the risk of all-cause and cause-specific mortality among surviving household members after a suicide within the household among members of the 100 Million Brazilian Cohort.

## Introduction

Suicide is a major global public health issue with profound impacts that extend beyond the deceased to surviving family members.^1,2,3,4^ The psychological, social, and economic consequences of suicide ripple through families and communities.^2,3,4^ Surviving family members are frequently confronted with a complex mix of grief, stigma, and structural vulnerability^5,6,7^ that adversely affects their mental^8,9,10,11^ and physical well-being.^9^ These challenges are heightened in low-income settings, where the burden of suicide intersects with barriers to mental health care, socioeconomic hardship, and cultural stigma.^12,13^

Research has largely focused on suicide risk among bereaved relatives,^2,14,15,16,17,18,19,20,21,22,23,24,25^ though less is known about how many people are exposed and need support in the broader household^19^ or about their overall mortality risk from nonsuicide causes.^9,26,27,28^ Prior studies have lacked unexposed comparison groups, reducing causal inference and limiting the ability to attribute observed outcomes to suicide exposure. Furthermore, there is a need to consider when excess mortality arises and how it varies over time following a suicide in the household. Most evidence comes from high-income countries,^2,9,14,15,16,17,18,19,20,21,22,23,24,26,27,28^ where more robust health systems may buffer harms. In contrast, household socioeconomic stressors and sociodemographic characteristics remain understudied in low-income settings where risks may be amplified.^17^

Understanding the long-term consequences of suicide exposure on mortality is essential for guiding public health and postvention efforts.^29,30^ However, few population-based studies have examined these outcomes.^17,19^ This study used nationwide administrative data from Brazil to investigate mortality outcomes among household members exposed to suicide in socioeconomically disadvantaged settings. Objectives were to (1) estimate the increased risk of all-cause and cause-specific mortality among household members exposed to suicide, (2) identify risk factors for subsequent suicide among these household members, and (3) assess timing of mortality after exposure. It was hypothesized that exposure to suicide increases mortality, with risks varying by timing, sociodemographic factors, and household conditions.

## Methods

### Ethical Considerations

Ethical approval was obtained from the Federal University of Bahia and Centro de Pesquisa Gonçalo Moniz, Fundação Oswaldo Cruz, Bahia. As no personally identifiable information was included, informed consent was waived. The study followed the Strengthening the Reporting of Observational Studies in Epidemiology (STROBE) reporting guideline.

### Study Design and Data Sources

This nationwide longitudinal study used data from the 100 Million Brazilian Cohort (100MCohort) linked with the Brazilian Mortality Information System (SIM) (2001-2018).^31^ The 100MCohort, established by the Centre for Data and Knowledge Integration for Health (CIDACS),^32^ involves linkage between the Unified Registry for Social Programs (CadÚnico) and Brazilian Health Information Systems.^33^ CadÚnico eligibility requires per capita monthly family income equal to half of the minimum wage or less.^34^ From 2011 to 2018, CadÚnico included 131 697 800 individuals, with higher proportions of young people, female individuals, and urban residents. SIM is a nationwide health information system for recording mortality data, encompassing all deaths in the country and their causes.^35^

### Data Linkage

Linking the 100MCohort baseline and SIM datasets (2001-2018) used a 2-step process based on 5 individual identifiers (name, date of birth, sex, mother’s name, and municipality) via the CIDACS record linkage tool.^36^ Exact matching was followed by similarity-score linkage for unmatched entries. Accuracy was assessed through manual validation of a random subset and receiver operating characteristic analysis, with sensitivity and specificity exceeding 92% (eFigure 1 and eTable 1 in Supplement 1).

### Study Population

This study included individuals aged 10 years or older at registration or who reached this age during follow-up (January 1, 2001, to December 31, 2018). Excluded individuals were (1) those aged above 110 years at registration and (2) individuals who registered in CadÚnico on the last day of the follow-up (December 31, 2018), died, or became exposed on the day of cohort enrollment (eFigure 2 in Supplement 1).

### Exposure and Follow-up

Suicide survivors were defined as household members of the decedent, given their exposure to a suicide and likelihood of being personally affected by it.^19,29^ In the 100MCohort, household members share a family and/or household code, and each individual has a unique identifier linked to socioeconomic and demographic characteristics. Using these codes, we identified all members of each household and the first suicide within it, referred to as the suicide index case. The exposed individuals were those who had a suicide index case in their household, identified by sharing the same household code. Unexposed individuals were classified as: (1) those who never had a suicide index case during the follow-up period or (2) those who had an suicide index case but were considered unexposed until the occurrence of that event.

Unexposed individuals were followed up from the time of their registration in the cohort baseline until 1 of the following occurrences: (1) death due to any cause (including suicide), (2) end of the follow-up (December 31, 2018), or (3) the date of the occurrence of the suicide index case within the same household. Exposed individuals were followed up from the occurrence of the suicide index case until 1 of the following: (1) death due to any cause (including suicide) or (2) end of the follow-up (December 31, 2018) (eFigure 3 in Supplement 1).

### Primary and Secondary Outcomes

The primary outcome was all-cause mortality, defined as death from any cause as classified by the *International Statistical Classification of Diseases and Related Health Problems, Tenth Revision (ICD-10)*.^35^ As suicide is an expected outcome in families with a history of suicide, the main analyses focused on all-cause mortality excluding suicide (*ICD-10* codes X60-X84) to avoid confounding. Separate analyses were conducted for all-cause mortality including suicide and for suicide-specific mortality to enable comparison and interpretation. Secondary outcomes included (1) other mortality due to external causes (*ICD-10* codes V00-Y99, excluding suicide); (2) the 5 most frequent specific causes of external mortality in our dataset: violence (X85-Y09), transport injuries (V01-V99), falls (W00-W19), accidental drowning (W65-W74), and accidental poisoning (X43); (3) mortality due to natural causes (all other causes excluding external causes); and (4) the most frequent specific natural causes of death, including diseases of the circulatory system (I00-I99), neoplasms (C00-D48), metabolic diseases (E70-E89), diseases of the respiratory system (J00-J99), and parasitic diseases (B65-B83).

### Statistical Analysis

For the first objective, sex-specific age-standardized rates were estimated using person-year as the denominator and using the Brazilian 2015 official population projection as the standard.^33^ A multivariable, time-varying Cox regression model was used to assess whether exposure to a household suicide was associated with subsequent mortality among surviving members. This approach considers changing variables and time-dependent covariates,^37^ allowing exposure to be treated dynamically; individuals contributed person-time as unexposed until a household suicide occurred, after which they were classified as exposed (eFigure 3 in Supplement 1). Crude and adjusted hazard ratios (HRs) were estimated with 95% CIs. Models were adjusted for confounders identified in prior literature, including sex, age, race, region, location of residence, urbanicity, unemployment, housing materials, water supply, sanitation, and waste. Race was derived from the CadÚnico database and self-reported as Asian, Black, Indigenous, Parda, or White. Parda (Portuguese for brown) denotes individuals of predominantly Black or mixed ancestry, including European, African, and Indigenous origins. The attributable risk percentage (%AR) was calculated using the Levin formula: %AR_exp = [(Incidence_exp – 1) ÷ (Incidence_exp – 1) + 1] × 100, to quantify the proportion of deaths attributable to exposure (exp).^38^

For the second objective, the focus was on individuals in households with a prior suicide. A multivariable Cox regression was used to examine factors associated with subsequent suicide or all-cause mortality. The final model, optimized through stepwise selection, included variables for (1) the sex and age of the index case and (2) the sex and age of surviving household members (measured at the time of the index case), as well as household conditions and geographic region. Household conditions were captured using a composite score from 0 to 4, where 0 indicates no access and 4 indicates full access to essential services (water supply, sanitation, adequate housing materials, and waste disposal). Two interaction terms (sex and age of the index case) were tested to evaluate whether the outcomes associated with other risk factors varied according to these characteristics. Details of model specification and covariates are provided in eAppendices 1, 2, 3, and 4 in Supplement 1.

Finally, the proportional mortality of subsequent deaths and suicide events by year of occurrence after the index case were calculated. Nearly half of these deaths occurred within the first 2 years, which guided the definition of the immediate period as 2 or fewer years and the distant period as 3 or more years. The analysis further assessed whether risk factors varied between immediate (≤2 years) and distant (≥3 years) follow-up periods by including multiplicative interaction terms between each covariate and a dichotomous variable indicating follow-up time. The model can be represented as follows: log[hazard(t)] = β_0_ + β_1_ (≥3 years period) + β_2_ (*characteristic X*) + β_3_ (≥3 years period × *characteristic X*), where *characteristic X* refers to an explanatory variable of interest, such as the sex of the index individual, age at death, or sex of surviving household members, among others. The interaction term (β_3_) allows us to assess whether the association of characteristic X with the risk of the outcome (all-cause mortality or suicide) differs between the period shortly after the death (≤2 years) and the later period (≥3 years). Although the model includes a single variable, multiple factors were included simultaneously, each with its respective main association and interaction term with time since death, allowing us to evaluate time-varying associations. The joint significance of the interaction terms was tested using the Wald test.

HRs and 95% CIs were reported separately for each time interval. All analyses were performed using Stata version 15.1 (StataCorp LLC). A 2-sided *P* < .05 was considered statistically significant.

To address potential bias from differences in follow-up time between exposed and unexposed groups, we performed sensitivity analyses using (1) a doubly robust approach adjusting the main analysis by adding exposure time as a covariate, (2) restricting follow-up to cohort entry for exposed and unexposed, and (3) simulating equal follow-up durations. For analyses of immediate and distant periods, we also tested alternative cutoffs and time scales (≤1 year vs ≥2 years; ≤3 years vs ≥4 years). Data were analyzed from March 2023 to September 2025.

## Results

The cohort consisted of 101 346 669 individuals from 26 594 713 households, with 167 475 individuals in the exposed group. There were 47 982 suicide index cases (eFigure 2 in Supplement 1). Compared with the nonexposed group, individuals exposed to suicide were more likely to be female (90 405 individuals [54%]), young people (aged 10-24 years: 111 791 individuals [67%]), Parda (82 407 individuals [53%]), and unemployed (162 916 individuals [97%]) (Table 1). The exposed group also included 11 070 (7%) Black and 57 726 (37%) White individuals.

**Table 1.** Description of Study Population by Exposure to an Index Suicide Case in the Same Household (N = 101 346 669)

Characteristic	Participants, No. (%)		*P* value^a^
	Nonexposure (n = 101 179 194)	Exposure (n = 167 475)	
Sex			
Male	47 658 742 (47)	77 070 (46)	<.001
Female	53 520 452 (53)	90 405 (54)	
Age cohort			
10-24 y	58 804 961 (58)	111 791 (67)	<.001
25-59 y	37 278 090 (37)	50 809 (30)	
60-110 y	5 096 143 (5.0)	4875 (2.9)	
Race^b^			
Asian descendants	372 702 (0.4)	506 (0.3)	<.001
Black	7 036 884 (7.5)	11 070 (7.1)	
Indigenous	538 453 (0.6)	3627 (2.3)	
Parda^c^	55 370 996 (59)	82 407 (53)	
White	31 068 881 (33)	57 726 (37)	
Unknown	6 791 278	12 139	
Region			
Northeast	40 065 572 (40)	61 754 (37)	<.001
North	10 465 502 (10)	16 459 (9.8)	
Southeast	32 322 337 (32)	45 742 (27)	
South	11 522 233 (11)	31 126 (19)	
Central-West	6 710 100 (6.6)	12 216 (7.3)	
Unknown	93 450	178	
Location residence			
Urban	73 144 808 (74)	113 151 (69)	<.001
Rural	25 327 550 (26)	51 598 (31)	
Unknown	2 706 836	2726	
Unemployed			
Yes	96 309 167 (95)	162 916 (97)	<.001
No	4 870 027 (4.8)	4559 (2.7)	
Construction materials			
Uninformed	3 630 860 (3.6)	3644 (2.2)	<.001
Bricks or cement	72 657 604 (72)	109 128 (65)	
Wood, vegetal materials, and other	24 890 730 (25)	54 703 (33)	
Sanitation			
Uninformed	4 301 559 (4.3)	4391 (2.6)	<.001
Public network	42 391 809 (42)	59 256 (35)	
Septic tank	14 932 195 (15)	27 551 (16)	
Homemade septic tank	24 675 659 (24)	45 457 (27)	
Ditch or other	14 877 972 (15)	30 820 (18)	
Water supply			
Uninformed	3 630 469 (3.6)	3634 (2.2)	<.001
Public network (running water)	68 163 602 (67)	107 549 (64)	
Well, natural sources, or other	29 385 123 (29)	56 292 (34)	
Waste			
Uninformed	3 630 901 (3.6)	3642 (2.2)	<.001
Public collection system	71 129 518 (70)	108 884 (65)	
Burned, buried, outdoor disposal, or other	26 418 775 (26)	54 949 (33)	

^a^ Pearson χ^2^ test.

^b^ Race was self-reported.

^c^ Parda, which translates from Portuguese as brown, is used to denote individuals whose racial background is predominantly Black and those with multiracial ancestry, including European, African, and Indigenous origins.

### Risk of All-Cause and Specific-Cause Mortality

**Figure 1.**
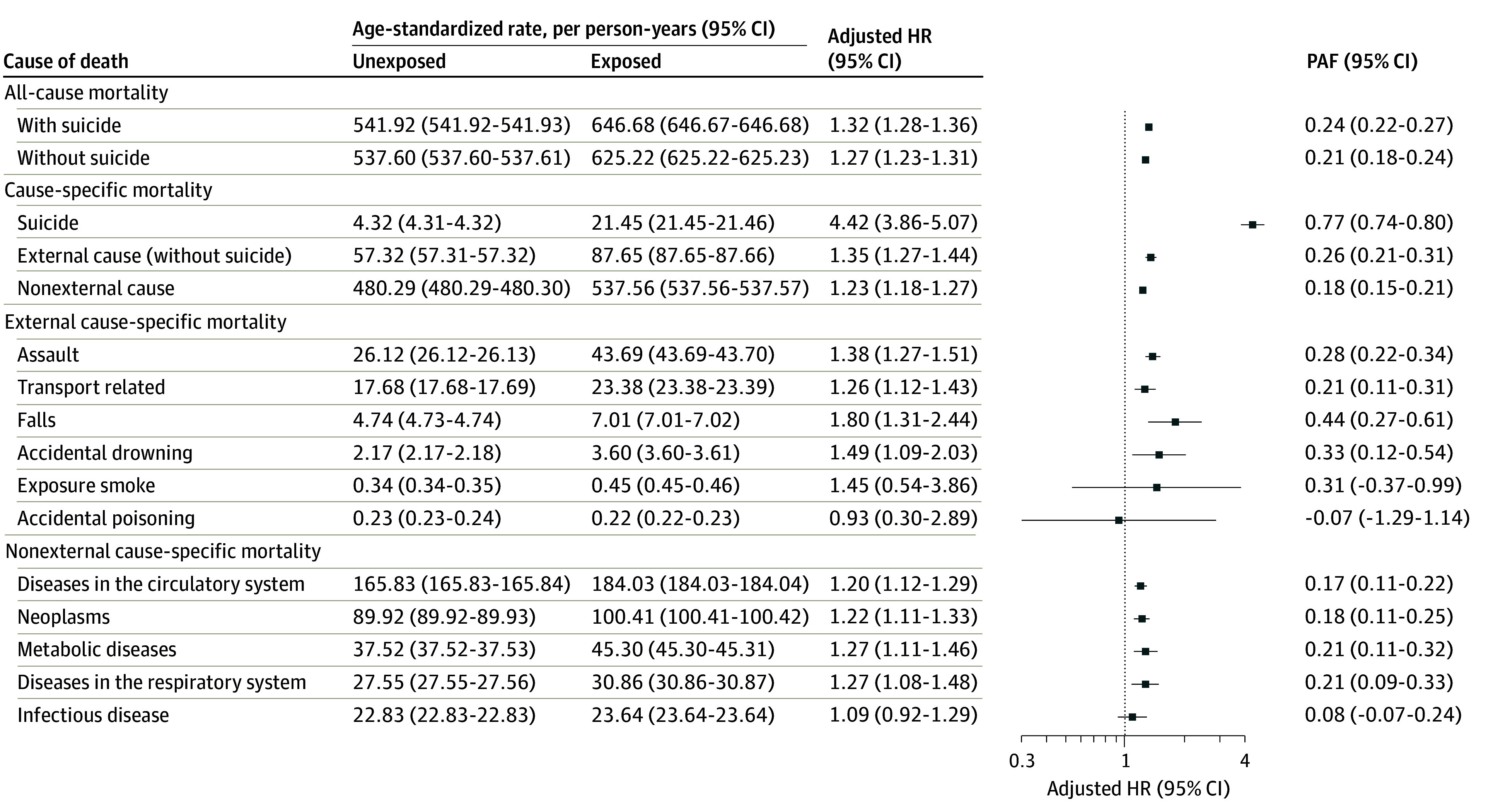
Cox Multivariate Model of the Association Between Suicide Index Case and the Risk of Mortality Model adjusted for sex, age cohort, race, region, location residence, unemployed, construction materials, water supply, and waste. HR indicates hazard ratio; PAF, population attributable fraction.

Exposure to a suicide index case was associated with a 32% higher risk of mortality when including suicide (HR, 1.32; 95% CI, 1.28-1.36), and 27% higher risk when excluding suicide (HR, 1.27; 95% CI, 1.23-1.31), compared with nonexposed individuals (Figure 1). Suicide-specific mortality risk was over 4 times higher among exposed household members (HR, 4.42; 95% CI, 3.86-5.07). Exposed members also had a higher risk of death from external causes (excluding suicide) (HR, 1.35; 95% CI, 1.27-1.44) and natural causes (HR, 1.23; 95% CI, 1.18-1.27). Among external causes, higher risks were observed for deaths due to falls (HR, 1.80; 95% CI, 1.31-2.44), drowning (HR, 1.49; 95% CI, 1.09-2.03), assault (HR, 1.38; 95% CI, 1.27-1.51), and transport crashes (HR, 1.26; 95% CI, 1.12-1.43). For natural causes, higher risks were found for circulatory diseases (HR, 1.20; 95% CI, 1.12-1.29), neoplasms (HR, 1.22; 95% CI, 1.11-1.33), metabolic conditions (HR, 1.27; 95% CI, 1.11-1.46), and respiratory diseases (HR, 1.27; 95% CI, 1.08-1.48), with no clear association for infectious diseases (HR, 1.09; 95% CI, 0.92-1.29) (Figure 1).

### Main Risk Factors Among Surviving Household Members

Exposure to a female index case was associated with higher all-cause mortality excluding suicide among surviving household members (HR, 1.27; 95% CI, 1.16-1.40) compared with a male index case, but not with higher suicide (HR, 1.39; 95% CI, 0.94-2.06). Exposure to a younger index case (aged 10-24 years) was associated with higher all-cause mortality (HR, 1.16; 95% CI, 1.08-1.26) and suicide (HR, 1.68; 95% CI, 1.22-2.31) compared with an older index case (aged 60 years or older). An interaction showed that a young female index case was associated with lower risk of all-cause mortality (HR, 0.72; 95% CI, 0.61-0.84) relative to older male cases (Table 2).

**Table 2.** Cox Regression Model by Characteristics of the Index Suicide Case, Sociodemographic Characteristics of the Members, and Household Conditions Among Individuals Who Have Experienced a Previous Suicide Within the Same Household, 2001 to 2018

Characteristic	All-cause mortality outcome (without suicide)		Suicide outcome	
	HR (95% CI)	Adjusted HR (95% CI)	HR (95% CI)	Adjusted HR (95% CI)
Characteristics of the index case				
Sex of the index case				
Male	1 [Reference]	1 [Reference]	1 [Reference]	1 [Reference]
Female	1.13 (1.05-1.22)	1.27 (1.16-1.40)	1.48 (1.12-1.96)	1.39 (0.94-2.06)
Age at death of the index case				
≥60 y	1 [Reference]	1 [Reference]	1 [Reference]	1 [Reference]
10-24 y	1.19 (1.11-1.27)	1.16 (1.08-1.26)	1.70 (1.30-2.21)	1.68 (1.22-2.31)
25-59 y	1.73 (1.55-1.94)	1.04 (0.92-1.19)	1.22 (0.69-2.16)	1.09 (0.54-2.17)
Sex of the index case and age at death of the index case				
Male ≥60 y	1 [Reference]	1 [Reference]	1 [Reference]	1 [Reference]
Female 10-24 y	0.64 (0.55-0.75)	0.72 (0.61-0.84)	0.57 (0.32-1.03)	0.70 (0.39-1.27)
Female 25-59 y	1.47 (1.12-1.96)	0.94 (0.72-1.24)	1.78 (0.52-6.16)	1.88 (0.54-6.51)
Sociodemographic characteristics of surviving members				
Sex of surviving household members				
Female	1 [Reference]	1 [Reference]	1 [Reference]	1 [Reference]
Male	1.50 (1.41-1.60)	1.75 (1.64-1.86)	3.54 (2.63-4.77)	3.58 (2.65-4.84)
Mortality age of the surviving household members at the date of occurrence of the Index case				
10-24 y	1 [Reference]	1 [Reference]	1 [Reference]	1 [Reference]
25-59 y	3.57 (3.29-3.89)	3.81 (3.51-4.15)	1.42 (1.09-1.85)	1.64 (1.25-2.14)
≥60 y	22.74 (20.84-24.82)	24.13 (22.09-26.37)	0.92 (0.47-1.82)	1.04 (0.52-2.06)
Household characteristics				
Living conditions^a^				
0	1 [Reference]	1 [Reference]	1 [Reference]	1 [Reference]
1	1.02 (0.94-1.11)	1.03 (0.94-1.12)	0.43 (0.29-0.65)	0.47 (0.31-0.71)
2	0.96 (0.87-1.05)	0.99 (0.90-1.10)	0.62 (0.43-0.91)	0.61 (0.42-0.89)
3	0.78 (0.70-0.86)	0.79 (0.71-0.88)	0.34 (0.23-0.51)	0.33 (0.22-0.50)
4	0.73 (0.66-0.81)	0.76 (0.69-0.85)	0.43 (0.30-0.62)	0.49 (0.33-0.73)
Region				
Northeast	1 [Reference]	1 [Reference]	1 [Reference]	1 [Reference]
North	0.85 (0.75-0.97)	1.09 (0.96-1.23)	1.51 (0.94-2.43)	1.22 (0.75-1.98)
Southeast	1.04 (0.96-1.12)	1.09 (1.00-1.18)	1.00 (0.68-1.47)	1.15 (0.76-1.73)
South	1.09 (1.01-1.19)	1.17 (1.07-1.28)	2.05 (1.45-2.88)	2.13 (1.51-3.01)
Central-West	0.97 (0.85-1.10)	1.09 (0.95-1.24)	2.80 (1.84-4.25)	2.89 (1.89-4.41)

Abbreviation: HR, hazard ratio.

^a^ Living conditions defined as household conditions measured using a composite score from 0 to 4, where 0 indicates no access and 4 indicates full access to essential services, including water supply, sanitation, adequate housing materials, and waste disposal.

Among surviving household members, male individuals had a 75% higher risk of all-cause mortality excluding suicide (HR, 1.75; 95% CI, 1.64-1.86) and over 3 times higher risk of suicide (HR, 3.58; 95% CI, 2.65-4.84) compared with female individuals. Compared with young survivors, risk of all-cause mortality was higher among those aged 25 to 59 years (HR, 3.81; 95% CI, 3.51-4.15) and those aged 60 years or older (HR, 24.13; 95% CI, 22.09-26.37), whereas suicide risk was higher among those aged 25 to 59 years (HR, 1.64; 95% CI, 1.25-2.14) but not among those aged 60 years or older (HR, 1.04; 95% CI, 0.52-2.06).

Better household conditions, reflected by having all 4 essential services, had lower risk of all-cause mortality (HR, 0.76; 95% CI, 0.69-0.85) and suicide (HR, 0.49; 95% CI, 0.33-0.73) compared with households without these essential services. Regionally, suicide risk was higher in the South (HR, 2.13; 95% CI, 1.51-3.01) and Central-West (HR, 2.89; 95% CI, 1.89-4.41) compared with the Northeast. For all-cause mortality excluding suicide, the South showed increased risk (HR, 1.17; 95% CI, 1.07-1.28) (Table 2).

### Timing of Subsequent Deaths After the Index Case: Comparison of Immediate (≤2 Years) and Distant Events (>3 Years)

**Figure 2.**
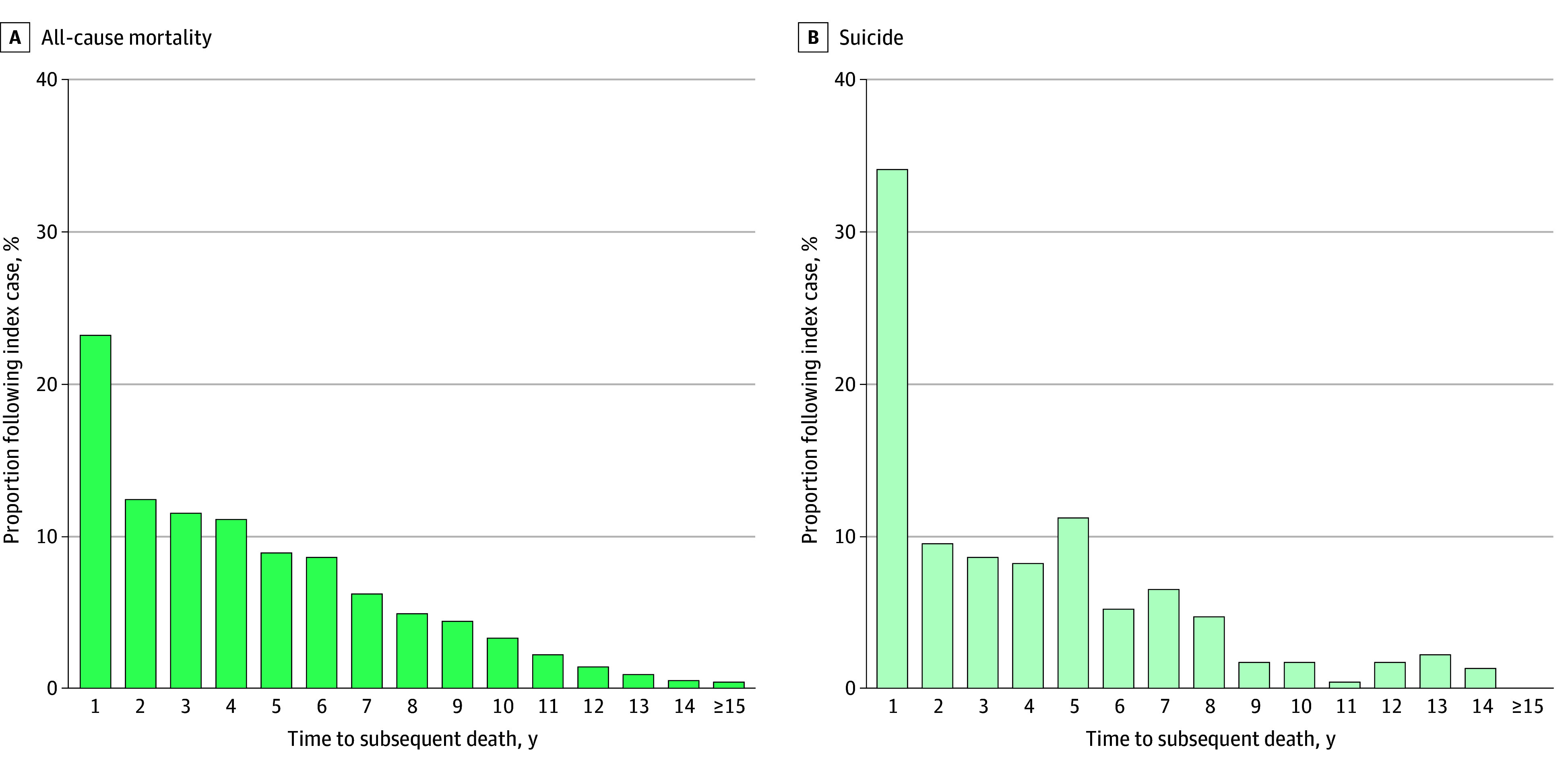
Proportion of Subsequent All-Cause Mortality (Excluding Suicide) and Suicide in Years All-cause mortality (4009 individuals) and suicide (232 individuals).

**Figure 3.**
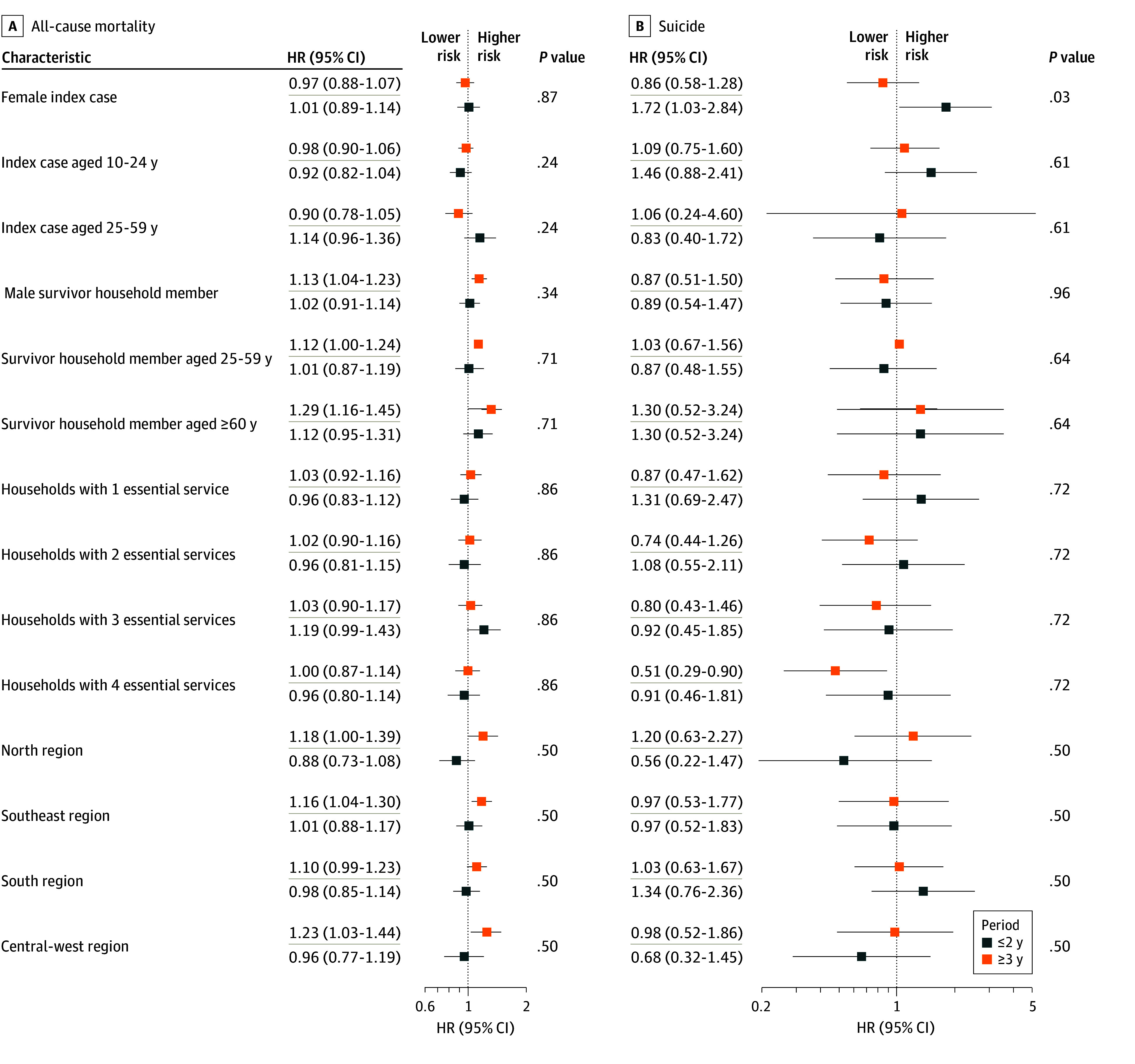
Factors Associated with Immediate (≤2 Years) and Distant (≥3 Years) Time-to-Event Mortality *P* value represents the test of interaction between the time since the index case's death (≤2 years vs ≥3 years) and the respective characteristic under evaluation. HR indicates hazard ratio.

Most subsequent deaths, both all-cause and by suicide, occurred within 2 years of the index suicide. Among all-cause mortality (excluding suicide; 4009 individuals), 1429 (35.6%) occurred within 2 years (931 [23.2%] in year 1; 498 [12.4%] in year 2) (Figure 2). Among suicides (232 individuals), 101 (43.6%) occurred in the same period (79 [34.1%] in year 1; 22 [9.5%] in year 2). For all-cause mortality, no differences were observed between immediate (≤2 years) and distant (≥3 years) periods. For suicide, a time-dependent association was found; during the first 2 years, having a female index case was associated with higher suicide risk among survivors (HR, 1.72; 95% CI, 1.03-2.84; *P* = .03), an outcome not observed after 3 years (HR, 0.86; 95% CI, 0.58-1.28) (Figure 3). All sensitivity analyses yielded results consistent with those of the main model (eTables 6-9, eFigure 5 in Supplement 1). A summary of key findings and associated risk factors is provided in eFigure 6 in Supplement 1.

## Discussion

This large-scale cohort study is the first we know of to examine timing and risk factors for all- and cause-specific mortality after household exposure to suicide. Exposure was associated with a 27% increased in all-cause mortality (excluding suicide) and over 4-fold higher suicide risk, with a population attributable fraction of 77%. Risks were elevated when the index case was younger or female, and when surviving household members were male, aged 25 to 59 years, or living in households with poor infrastructure. Over half of subsequent suicides occurred within the first 2 years, reinforcing the urgency of targeted early interventions (eFigure 7 in Supplement 1).

Prior research shows elevated suicide risk among individuals with a family history of suicide,^2,14,15,16,17,18,19,20,21,22,23,24,25^ yet broader mortality outcomes remain underexplored.^9,26,27,28^ In Taiwan, higher rates of suicide (rate ratio [RR], 4.61; 95% CI, 4.02-5.29) and accidental deaths (RR, 1.62; 95% CI, 1.43-1.84) were reported among first-degree relatives of suicide decedents.^27^ Another study found increased risks of suicide (HRs up to 15.67; 95% CI, 2.09-117.41), homicide (HRs up to 23.26; 95% CI, 3.10-174.56), and dementia (HR, 4.41; 95% CI, 1.14-17.05) in suicide-exposed individuals, but not for all-cause mortality relative to unnatural deaths (HR, 0.95; 95% CI, 0.87-1.04).^28^ The current study expands on existing evidence, showing that household suicide exposure is linked to higher risks of both suicide and all-cause mortality, including from metabolic, respiratory, circulatory diseases, and neoplasms. All major external and natural causes of death were elevated except parasitic diseases, which are likely more tied to environmental and socioeconomic factors than psychosocial stressors. The 442% increased risk of suicide observed in this study exceeds estimates from high-income countries such as Denmark (RR, 2.58; 95% CI, 1.84-3.61)^21^ and South Korea (RR, 2.75; 95% CI, 2.55-2.97),^16^ suggesting greater impact in settings characterized by heightened social and structural vulnerability.

The mechanisms linking family suicide history and increased mortality likely involve multiple pathways.^9,26,27,28^ Suicide exposure is traumatic and often results in grief, guilt, stigma, and family disruption.^5,6,7^ These factors are associated with suicide behaviors,^2,14,15,16,17,18,19,20,21,22,23,24,25^ and contribute to depression, anxiety, PTSD, and substance use,^3,4,5,6,7,8,9,10,11,18^ thereby leading to early death. Chronic stress may disrupt the hypothalamic-pituitary-adrenal axis, suppress immune function, and increase the risk of cardiovascular and metabolic disorders,^39^ explaining elevated deaths from circulatory, respiratory, and metabolic diseases. Social and economic stressors may also play a role.^2,18^ Suicide loss can trigger financial hardship, social withdrawal, family conflict, and caregiving burdens.^5,6,7^ Male survivors and adults aged 25 to 59 years were especially affected, suggesting that adult men may face distinct challenges in coping with household suicide. Death of a younger index case (aged 10-24 years) also resulted in higher mortality risk, reflecting the destabilizing impact of an unexpected death and the profound grief it provokes.^20,21,40^

Over a third of all-cause deaths (35.6%) and nearly half of suicides (43.6%) occurred within 2 years of the index suicide. In the immediate aftermath of the suicide, family members often experience intense grief marked by guilt, blame, and stigma,^2,5,6,7,18^ leading to enduring physical, psychological, and psychosomatic difficulties.^18^ These challenges highlight the urgent need for family-centered interventions after suicide loss.^12^ Although the bereaved may express a notable desire to participate in suicide support groups,^2,18^ access to mental health services or specialized care for survivors remains scarce, especially in lower-resource contexts such as Brazil.^41^ In the current study, survivors living in better housing conditions showed lower mortality risk, reinforcing the role of socioeconomic factors in mitigating outcomes after suicide loss.^42,43^

### Strengths and Limitations

Previous studies have been limited to high-income settings with small samples and lacked population-based comparison groups. This study leveraged a large population-based cohort to overcome these gaps. This design provides sufficient power to examine rare outcomes and exposures, including suicide occurrence and recurrence within households. Most earlier studies used case-control designs, which precluded estimation of population-level measures such as suicide, relative risks, and attributable risks. Other studies focused only on individuals with recorded deaths, limiting comparisons by family suicide history. The current approach enabled the estimation of these rates and risks and the assessment of the interval between an index suicide and subsequent suicide within the same household, offering important insights for prevention strategies.

Some limitations are unmeasured confounders that could introduce bias and the lack of information regarding parental status, as well as access to mental health care. It is noteworthy that some causes of death, such as ill-defined or unknown causes, events of undetermined intent, and accidental deaths, may conceal hidden suicides, potentially influencing the findings. Moreover, this study population was composed of low-income individuals, limiting generalizability. Additionally, suicide may be underregistered; however, in Brazil, external causes of death undergo systematic review by medical-legal institutes, and the SIM database is recognized for its high quality, minimizing misclassification.^44^

## Conclusions

This cohort study of 101 346 669 individuals in Brazil found that household exposure to suicide resulted in over 4-fold increase in subsequent suicide risk and a 27% rise in all-cause mortality (excluding suicide) among surviving members (eFigure 7 in the Supplement). These far-reaching health consequences, associated with external and nonexternal causes, were most pronounced in the first 2 years, highlighting the urgency for early intervention. These findings call for integrated postvention strategies including bereavement care, psychosocial support, and clinical follow-up, especially in lower-resource settings, to advance global mental health equity and support Sustainable Development Goal 3.4: “reducing premature mortality from noncommunicable diseases and promoting mental well-being.”^45^

## Notes

Supplement 1. eAppendix 1. Data Sources and Linkage of the Datasets eTable 1. Accuracy Analysis of the Linkage Between CadUnico and Mortality Information System in a Sample of 10 000 Record Pairs eFigure 1. ROC Curve of the Linkage Between Mortality and CadUnico From 2001 to 2015 eFigure 2. Flowchart of the Selected Population eTable 2. Summary Measures of the Variable Number of People in the Household eTable 3. Descriptive Analysis by Index Case eAppendix 2. Statistical Modeling eTable 4. Test of Proportional-Hazards Assumption eFigure 3. Follow-Up eFigure 4. Log-Log Survival Probability eTable 5. All-Cause Mortality and Suicide Rates by Characteristics of the Index Suicide Case Among Individuals Who Have Experienced a Previous Suicide Within the Same Household, 2001 to 2018 eAppendix 3. Sensitivity Analyses eTable 6. Hazard Ratios for Suicide and All-Cause Mortality Including Time-Dependent Exposure and Duration of Follow-Up eTable 7. Hazard Ratios for Suicide and All-Cause Mortality Using a Common Definition of Follow-up Entry for Exposed and Unexposed eTable 8. Distribution of Follow-Up Time (Years) by Definition of Baseline Date and Exposure Status eTable 9. Hazard Ratios for Suicide and All-Cause Mortality Using Common Follow-up Windows for Exposed and Unexposed eFigure 5. Factors Associated with Immediate (≤1 year), Intermediate (2-4 years), and Distant (≥5 years) Deaths by All-Cause Mortality (Excluding Suicide) and Suicide, 2001 to 2018 eFigure 6. Summary of Statistically Significant Risk and Protective Factors for All-Cause Mortality (Excluding Suicide) and Suicide eAppendix 4. Pathways Linking Household Suicide Exposure to Subsequent Mortality and Target Groups for Postvention Interventions eFigure 7. Pathways Linking Household Suicide Exposure to Subsequent Mortality and Targets for Postvention Interventions eReferences

Supplement 2. Data Sharing Statement

## References

[1] World Health Organization. World health statistics 2019: monitoring health for the SDGs, sustainable development goals. Accessed April 2, 2024. https://www.who.int/publications-detail-redirect/9789241565707

[2] Spillane A, Larkin C, Corcoran P, Matvienko-Sikar K, Riordan F, Arensman E. Physical and psychosomatic health outcomes in people bereaved by suicide compared to people bereaved by other modes of death: a systematic review. BMC Public Health. 2017;17(1):939. doi:10.1186/s12889-017-4930-3 29228916 PMC5725957

[3] Brent D, Melhem N, Donohoe MB, Walker M. The incidence and course of depression in bereaved youth 21 months after the loss of a parent to suicide, accident, or sudden natural death. Am J Psychiatry. 2009;166(7):786-794. doi:10.1176/appi.ajp.2009.08081244 19411367 PMC2768496

[4] Levi-Belz Y, Gilo T. Emotional distress among suicide survivors: the moderating role of self-forgiveness. Front Psychiatry. 2020;11:341. doi:10.3389/fpsyt.2020.00341 32390889 PMC7190787

[5] Tal Young I, Iglewicz A, Glorioso D, . Suicide bereavement and complicated grief. Dialogues Clin Neurosci. 2012;14(2):177-186. doi:10.31887/DCNS.2012.14.2/iyoung 22754290 PMC3384446

[6] Mitchell AM, Kim Y, Prigerson HG, Mortimer MK. Complicated grief and suicidal ideation in adult survivors of suicide. Suicide Life Threat Behav. 2005;35(5):498-506. doi:10.1521/suli.2005.35.5.498 16268767

[7] Mitchell AM, Kim Y, Prigerson HG, Mortimer-Stephens M. Complicated grief in survivors of suicide. Crisis. 2004;25(1):12-18. doi:10.1027/0227-5910.25.1.12 15384652

[8] Mitchell AM, Terhorst L. PTSD symptoms in survivors bereaved by the suicide of a significant other. J Am Psychiatr Nurses Assoc. 2017;23(1):61-65. doi:10.1177/1078390316673716 27742751

[9] Erlangsen A, Runeson B, Bolton JM, . Association between spousal suicide and mental, physical, and social health outcomes: a longitudinal and nationwide register-based study. JAMA Psychiatry. 2017;74(5):456-464. doi:10.1001/jamapsychiatry.2017.0226 28329305 PMC5470398

[10] Bolton JM, Au W, Leslie WD, . Parents bereaved by offspring suicide: a population-based longitudinal case-control study. JAMA Psychiatry. 2013;70(2):158-167. doi:10.1001/jamapsychiatry.2013.275 23229880

[11] Ljung T, Sandin S, Långström N, Runeson B, Lichtenstein P, Larsson H. Offspring death and subsequent psychiatric morbidity in bereaved parents: addressing mechanisms in a total population cohort. Psychol Med. 2014;44(9):1879-1887. doi:10.1017/S0033291713002572 24176129

[12] World Health Organization. Preventing suicide: a global imperative. Accessed April 2, 2024. https://www.who.int/publications-detail-redirect/9789241564779

[13] Lichtenthal WG, Roberts KE, Donovan LA, . Investing in bereavement care as a public health priority. Lancet Public Health. 2024;9(4):e270-e274. doi:10.1016/S2468-2667(24)00030-6 38492580 PMC11110717

[14] Kõlves K, Milner A, Värnik P. Suicide rates and socioeconomic factors in Eastern European countries after the collapse of the Soviet Union: trends between 1990 and 2008. Sociol Health Illn. 2013;35(6):956-970. doi:10.1111/1467-9566.12011 23398609

[15] Kõlves K, Zhao Q, Ross V, Hawgood J, Spence SH, de Leo D. Suicide and sudden death bereavement in Australia: a longitudinal study of family members over 2 years after death. Aust N Z J Psychiatry. 2020;54(1):89-98. doi:10.1177/0004867419882490 31647307

[16] Jang J, Park SY, Kim YY, . Risks of suicide among family members of suicide victims: a nationwide sample of South Korea. Front Psychiatry. 2022;13:995834. doi:10.3389/fpsyt.2022.995834 36311502 PMC9614235

[17] Calderaro M, Baethge C, Bermpohl F, Gutwinski S, Schouler-Ocak M, Henssler J. Offspring’s risk for suicidal behaviour in relation to parental death by suicide: systematic review and meta-analysis and a model for familial transmission of suicide. Br J Psychiatry. 2022;220(3):121-129. doi:10.1192/bjp.2021.158 35049479

[18] Spillane A, Matvienko-Sikar K, Larkin C, Corcoran P, Arensman E. What are the physical and psychological health effects of suicide bereavement on family members? An observational and interview mixed-methods study in Ireland. BMJ Open. 2018;8(1):e019472. doi:10.1136/bmjopen-2017-019472 29331974 PMC5781012

[19] Andriessen K, Rahman B, Draper B, Dudley M, Mitchell PB. Prevalence of exposure to suicide: a meta-analysis of population-based studies. J Psychiatr Res. 2017;88:113-120. doi:10.1016/j.jpsychires.2017.01.017 28199930

[20] Qin P, Mortensen PB. The impact of parental status on the risk of completed suicide. Arch Gen Psychiatry. 2003;60(8):797-802. doi:10.1001/archpsyc.60.8.797 12912763

[21] Qin P, Agerbo E, Mortensen PB. Suicide risk in relation to family history of completed suicide and psychiatric disorders: a nested case-control study based on longitudinal registers. Lancet. 2002;360(9340):1126-1130. doi:10.1016/S0140-6736(02)11197-4 12387960

[22] Runeson B, Asberg M. Family history of suicide among suicide victims. Am J Psychiatry. 2003;160(8):1525-1526. doi:10.1176/appi.ajp.160.8.1525 12900320

[23] Agerbo E. Risk of suicide and spouse’s psychiatric illness or suicide: nested case-control study. BMJ. 2003;327(7422):1025-1026. doi:10.1136/bmj.327.7422.1025 14593038 PMC261658

[24] Agerbo E. Midlife suicide risk, partner’s psychiatric illness, spouse and child bereavement by suicide or other modes of death: a gender specific study. J Epidemiol Community Health. 2005;59(5):407-412. doi:10.1136/jech.2004.024950 15831691 PMC1733072

[25] Tidemalm D, Runeson B, Waern M, . Familial clustering of suicide risk: a total population study of 11.4 million individuals. Psychol Med. 2011;41(12):2527-2534. doi:10.1017/S0033291711000833 21733212 PMC3207221

[26] Rostila M, Saarela J, Kawachi I. The forgotten griever: a nationwide follow-up study of mortality subsequent to the death of a sibling. Am J Epidemiol. 2012;176(4):338-346. doi:10.1093/aje/kws163 22814369

[27] Chen YY, Gunnell D, Wu CK, Hu YH, Lee PC. All-cause and cause-specific mortality in parents after the death of a child in Taiwan: a population-based cohort study. Psychosom Med. 2023;85(3):221-230. doi:10.1097/PSY.0000000000001182 36917483

[28] Ho HY, Chen YL. Causes of death in individuals exposed to spousal, parental, and child suicide: a nationwide population-based cohort comparison study. Eur J Epidemiol. 2023;38(11):1165-1174. doi:10.1007/s10654-023-01055-8 37843745

[29] Jordan JR. Lessons learned: forty years of clinical work with suicide loss survivors. Front Psychol. 2020;11:766. doi:10.3389/fpsyg.2020.00766 32411052 PMC7201040

[30] Jordan JR. Postvention is prevention—the case for suicide postvention. Death Stud. 2017;41(10):614-621. doi:10.1080/07481187.2017.1335544 28557579

[31] Barreto ML, Ichihara MY, Pescarini JM, . Cohort profile: the 100 Million Brazilian Cohort. Int J Epidemiol. 2022;51(2):e27-e38. doi:10.1093/ije/dyab213 34922344 PMC9082797

[32] Barreto ML, Ichihara MY, Almeida BA, . The Centre for Data and Knowledge Integration for Health (CIDACS): linking health and social data in Brazil. Int J Popul Data Sci. 2019;4(2):1140. 34095542 10.23889/ijpds.v4i2.1140 PMC8142622

[33] Informações de Saúde (TABNET). DATASUS. Accessed April 3, 2024. https://datasus.saude.gov.br/informacoes-de-saude-tabnet/

[34] VIS DATA 3 beta. Accessed April 3, 2024. https://aplicacoes.cidadania.gov.br/vis/data3/v.php?q[]=r6JtZI%2B0gbBtxKW25rV%2FfmdhhJFkl21kmK19Zm11ZmqmaX7KmZO20qfOnJm%2B6IianbSon7Stv8OcaJLHlNawmJi2wKmpa5Rwr2%2BGf2uMvNSn06qU0eTDlZuulqfipbavqozH0JXcoqa83bOTn62jrehrfX9naL3Cn92ibtHtwpl3g5ub5ayyiXKgzM5W5V5bnd2zk628mZnfmrp9d4yNl2mjbme84MCopbWWruKvrq2djsTKn9OeprzrvJauraiZ25%2BssZybytBlmm5jl7W7qamtp6PcYnd%2FZ1143m7Qnp%2FQ4IianbSon7R0s6%2BjoLycbt2yoNnMwpWqvJ6e2p2ybpuSd6eU1wDgyeSup1yqmqjen7axoI67wqaKrZjJ6m12q7Som5l%2Frrv62sPKlI2CptHkupWwsaubmZ2ybn2OxCTg1qaU0Judo566mq2ZZm2RnJvK0FOzf3qim39kbXhYit6rsLOloczCn4qhmH3evJahuqmv65ptspigd6eU1wDgyeSup1yqmqjen7axoPD405zLsFPB6m2Efo6xqrQ%3D

[35] World Health Organization. International Statistical Classification of Diseases, Tenth Revision (ICD-10). World Health Organization; 1992.

[36] Barbosa GCG, Ali MS, Araujo B, . CIDACS-RL: a novel indexing search and scoring-based record linkage system for huge datasets with high accuracy and scalability. BMC Med Inform Decis Mak. 2020;20(1):289. doi:10.1186/s12911-020-01285-w 33167998 PMC7654019

[37] Zhang Z, Reinikainen J, Adeleke KA, Pieterse ME, Groothuis-Oudshoorn CGM. Time-varying covariates and coefficients in Cox regression models. Ann Transl Med. 2018;6(7):121. doi:10.21037/atm.2018.02.12 29955581 PMC6015946

[38] Mansournia MA, Altman DG. Population attributable fraction. BMJ. 2018;360:k757. doi:10.1136/bmj.k757 29472187

[39] Sic A, Cvetkovic K, Manchanda E, Knezevic NN. Neurobiological implications of chronic stress and metabolic dysregulation in inflammatory bowel diseases. Diseases. 2024;12(9):220. doi:10.3390/diseases12090220 39329889 PMC11431196

[40] Rostila M, Saarela J, Kawachi I. Suicide following the death of a sibling: a nationwide follow-up study from Sweden. BMJ Open. 2013;3(4):e002618. doi:10.1136/bmjopen-2013-002618 23624991 PMC3641510

[41] Oliveira Alves FJ, Fialho E, Paiva de Araújo JA, . The rising trends of self-harm in Brazil: an ecological analysis of notifications, hospitalisations, and mortality between 2011 and 2022. Lancet Reg Health Am. 2024;31:100691. doi:10.1016/j.lana.2024.100691 38500959 PMC10945432

[42] Machado DB, Williamson E, Pescarini JM, . Relationship between the Bolsa Família national cash transfer programme and suicide incidence in Brazil: a quasi-experimental study. PLoS Med. 2022;19(5):e1004000. doi:10.1371/journal.pmed.1004000 35584178 PMC9162363

[43] Alves FJO, Machado DB, Barreto ML. Effect of the Brazilian cash transfer programme on suicide rates: a longitudinal analysis of the Brazilian municipalities. Soc Psychiatry Psychiatr Epidemiol. 2019;54(5):599-606. doi:10.1007/s00127-018-1627-6 30456426

[44] Rebouças P, Alves FJ, Ferreira A, . Avaliação da qualidade do Sistema Brasileiro de Informações sobre Mortalidade (SIM): uma scoping review. Cien Saude Colet. 2025;30(1):e08462023. doi:10.1590/1413-81232025301.08462023 39879455

[45] United Nations Department of Economic and Social Affairs. Goal 3: Ensure healthy lives and promote well-being for all at all ages. Accessed October 31, 2025. https://sdgs.un.org/goals/goal3

