# Supplementary material for: Mortality Risk Following a Household Suicide
Source: JAMA Netw Open. 2025 Nov 25;8(11):e2545286. doi: 10.1001/jamanetworkopen.2025.45286 (PMC12648350; doi:10.1001/jamanetworkopen.2025.45286)
Supplement: Supplement 1. — eAppendix 1. Data Sources and Linkage of the Datasets eTable 1. Accuracy Analysis of the Linkage Between CadUnico and Mortality Information System in a Sample of 10 000 Record Pairs eFigure 1. ROC Curve of the Linkage Between Mortality and CadUnico From 2001 to 2015 eFigure 2. Flowchart of the Selected Population eTable 2. Summary Measures of the Variable Number of People in the Household eTable 3. Descriptive Analysis by Index Case eAppendix 2. Statistical Modeling eTable 4. Test of Proportional-Hazards Assumption eFigure 3. Follow-Up eFigure 4. Log-Log Survival Probability eTable 5. All-Cause Mortality and Suicide Rates by Characteristics of the Index Suicide Case Among Individuals Who Have Experienced a Previous Suicide Within the Same Household, 2001 to 2018 eAppendix 3. Sensitivity Analyses eTable 6. Hazard Ratios for Suicide and All-Cause Mortality Including Time-Dependent Exposure and Duration of Follow-Up eTable 7. Hazard Ratios for Suicide and All-Cause Mortality Using a Common Definition of Follow-up Entry for Exposed and Unexposed eTable 8. Distribution of Follow-Up Time (Years) by Definition of Baseline Date and Exposure Status eTable 9. Hazard Ratios for Suicide and All-Cause Mortality Using Common Follow-up Windows for Exposed and Unexposed eFigure 5. Factors Associated with Immediate (≤1 year), Intermediate (2-4 years), and Distant (≥5 years) Deaths by All-Cause Mortality (Excluding Suicide) and Suicide, 2001 to 2018 eFigure 6. Summary of Statistically Significant Risk and Protective Factors for All-Cause Mortality (Excluding Suicide) and Suicide eAppendix 4. Pathways Linking Household Suicide Exposure to Subsequent Mortality and Target Groups for Postvention Interventions eFigure 7. Pathways Linking Household Suicide Exposure to Subsequent Mortality and Targets for Postvention Interventions eReferences [file jamanetwopen-e2545286-s001.pdf]

# Supplemental Online Content

Alves F, Rodrigues ES, Toledo L, et al. Mortality risk following a household suicide. *JAMA Netw Open*. 2025;8(11):e2545286. doi:10.1001/jamanetworkopen.2025.45286

eAppendix 1. Data Sources and Linkage of the Datasets
eTable 1. Accuracy Analysis of the Linkage Between CadUnico and Mortality Information System in a Sample of 10 000 Record Pairs
eFigure 1. ROC Curve of the Linkage Between Mortality and CadUnico From 2001 to 2015
eFigure 2. Flowchart of the Selected Population
eTable 2. Summary Measures of the Variable Number of People in the Household
eTable 3. Descriptive Analysis by Index Case
eAppendix 2. Statistical Modeling
eTable 4. Test of Proportional-Hazards Assumption
eFigure 3. Follow-Up
eFigure 4. Log-Log Survival Probability
eTable 5. All-Cause Mortality and Suicide Rates by Characteristics of the Index Suicide Case Among Individuals Who Have Experienced a Previous Suicide Within the Same Household, 2001 to 2018
eAppendix 3. Sensitivity Analyses
eTable 6. Hazard Ratios for Suicide and All-Cause Mortality Including Time-Dependent Exposure and Duration of Follow-Up
eTable 7. Hazard Ratios for Suicide and All-Cause Mortality Using a Common Definition of Follow-up Entry for Exposed and Unexposed
eTable 8. Distribution of Follow-Up Time (Years) by Definition of Baseline Date and Exposure Status
eTable 9. Hazard Ratios for Suicide and All-Cause Mortality Using Common Follow-up Windows for Exposed and Unexposed
eFigure 5. Factors Associated with Immediate (≤1 year), Intermediate (2-4 years), and Distant (≥5 years) Deaths by All-Cause Mortality (Excluding Suicide) and Suicide, 2001 to 2018
eFigure 6. Summary of Statistically Significant Risk and Protective Factors for All-Cause Mortality (Excluding Suicide) and Suicide
eAppendix 4. Pathways Linking Household Suicide Exposure to Subsequent Mortality and Target Groups for Postvention Interventions
eFigure 7. Pathways Linking Household Suicide Exposure to Subsequent Mortality and Targets for Postvention Interventions
eReferences

This supplemental material has been provided by the authors to give readers additional information about their work.

## Data Sources and Linkage of the Datasets

## 1. The Cadastro Único (CadÚnico) Database and the 100 Million Brazilian Cohort

The 100 Million Brazilian Cohort is derived from baseline data collected from families between January 1, 2001, and December 31, 2018, who registered to benefit from the Brazilian government's social programs through the Unified Registry for Social Programs (Cadastro Único para Programas Sociais – CadÚnico). This administrative database allows Brazilians to register personal information (such as age, sex, self-identified race/ethnicity, education, etc.) and household information (including household density, structural characteristics of the residence, etc.). The person responsible for the household registration must be at least 16 years old and is responsible for registering the entire family, regardless of the ages of other family members. Registration is allowed provided that certain criteria are met: (i) families with a monthly per capita income of up to half a minimum wage, (ii) families with a total monthly income of up to three minimum wages, (iii) families with income above three minimum wages but linked to social programs at the federal, state, or municipal levels, (iv) sole residents of a household, or (v) individuals experiencing homelessness.

Upon registration, individuals are assigned a unique identifier and their socioeconomic characteristics are recorded. By the end of 2017, CadÚnico encompassed around 131 million individuals, representing approximately 50% of Brazil's population. This tool plays a crucial role in identifying and characterizing low-income families, enabling the government to understand the socioeconomic conditions of the most disadvantaged populations and to select participants for social programs.

The 100 Million Brazilian Cohort also integrates health-related databases. For example, data identifying suicide cases were obtained from the Mortality Information System (Sistema de Informação sobre Mortalidade - SIM). Established by the Center for Integration of Data and Knowledge for Health/Oswaldo Cruz Foundation (CIDACS/FIOCRUZ), the cohort facilitates research and ongoing evaluation of social determinants and the impact of social policies and programs on health outcomes in Brazil. The cohort includes detailed demographic and socioeconomic information at both the individual and family/household levels. This comprehensive approach enables the identification of both families and each individual within the cohort.

Data linkage within the cohort is achieved using specialized codes and algorithms designed to connect databases efficiently and accurately through five identifiers: date of birth, municipality of residence, sex, individual name, and mother's name. The linkage process involves a two-step approach using the CIDACS-RL (Centro de Integração de Dados e Conhecimentos para Saúde – Record Linkage) system (https://gitHub.com/gcgbarbosa/cidacs-rl). Initially, deterministic linkage is performed. For records not linked deterministically, a similarity score ranging from 0 to 1 is calculated for all pairwise comparisons, and pairs with the highest similarity scores are considered linked. The quality of these linkages between CadÚnico and SIM has been thoroughly evaluated and validated.

## 2. Linkage and Accuracy Analysis of the Linkage of the Dataset

The linkage between the 100 Million Brazilian Cohort baseline and SIM datasets (2001-2018) occurred through a two-step process based on five individual-level identifiers (name, date of birth, sex, name of the mother, and municipality of residence) using the CIDACS record linkage tool. Initially, entries were linked based on exact matching. Subsequently, entries that did not achieve deterministic linkage were connected based on a similarity score calculated for pairwise comparisons. This score ranged from 0 to 1, with the highest similarity scores indicating linked pairs. Extensive evaluations and validation have been conducted to ensure the quality of linkages between the 100 Million Brazilian Cohort and SIM (for all causes of mortality).

The primary objective of the accuracy analysis is to assess the quality of the linkage based on the similarity between records. This step is performed after the linkage process has been completed and validated. It involves obtaining a sample of records stratified by three score ranges: high (above 0.95),

© 2025 Alves F et al. *JAMA Network Open*.

intermediate (0.90 to 0.95), and low (below 0.90). Records with scores greater than or equal to 0.95 but less than 1 are included in the high score range.

Each pair of records was manually evaluated and classified as either a true pair or a false pair. Following this, a Receiver Operating Characteristic (ROC) curve is constructed to determine the optimal cut-off point, which balances sensitivity (the proportion of true pairs correctly identified during manual verification) and specificity (the proportion of false pairs correctly identified during manual verification). Records with scores above or equal to this cut-off point are classified as linked, while those with scores below are classified as unlinked.In cases where sensitivity values are set to 100%, it indicates that all pairs of records classified as true during manual verification had scores above or equal to the optimal cut-off point identified by the ROC curve.

From the ROC curve, the optimal cut-off point of 0.92 (ROC curve area [Sensitivity/Specificity]: 0.923 [0.983/0.949]) was identified. Using this optimal cut-off to declare matches, 97.8% of the linked pairs were estimated to be true matches, and 2.2% of the linked pairs were estimated to be false matches. An estimated 5% of the true matches were not linked (eFigure 1, eTable 1).

© 2025 Alves F et al. *JAMA Network Open*.

eTable 1. Accuracy analysis of the linkage between CadUnico and Mortality Information System in a sample of 10,000 record pairs.

| **Cut-off point** | **Specificity; sensitivity** | **Total matches (%)** | **True matches (% of linked cases)** | **False matches (% of linked cases)** | **Lost Matches (% of true matches)** |
|---|---|---|---|---|---|
| ≥0.83 | SP=0.459; S=0.996 | 7551 (75.5) | 4686 (62.1) | 2865 (37.9) | 17 (0.4) |
| ≥0.84 | SP=0.576; S=0.993 | 6916 (69.2) | 4668 (67.5) | 2248 (32.5) | 35 (0.7) |
| ≥0.85 | SP=0.692; S=0.990 | 6290 (62.9) | 4657 (74.0) | 1633 (26.0) | 46 (1.1) |
| ≥0.86 | SP=0.789; S=0.987 | 5759 (57.6) | 4641 (80.6) | 1118 (19.4) | 62 (1.3) |
| ≥0.87 | SP=0.863; S=0.977 | 5322 (53.2) | 4595 (86.3) | 727 (13.7) | 108 (2.3) |
| ≥0.88 | SP=0.908; S=0.973 | 5060 (50.6) | 4575 (90.4) | 485 (9.6) | 128 (2.7) |
| ≥0.89 | SP=0.941; S=0.969 | 4872 (48.7) | 4557 (93.5) | 315 (6.5) | 146 (3.1) |
| ≥0.90 | SP=0.961; S=0.964 | 4741 (47.4) | 4534 (95.6) | 207 (4.4) | 169 (3.6) |
| ≥0.91 | SP=0.974; S=0.956 | 4638 (46.4) | 4498 (97.0) | 140 (3.0) | 205 (4.4) |
| ≥0.92 | SP=0.981; S=0.950 | 4570 (45.7) | 4470 (97.8) | 100 (2.2) | 233 (5.0) |
| ≥0.93 | SP=0.986; S=0.940 | 4495 (45.0) | 4423 (98.4) | 72 (1.6) | 280 (6.0) |
| ≥0.94 | SP=0.989; S=0.919 | 4381 (43.8) | 4323 (98.7) | 58 (1.3) | 380 (8.0) |
| ≥0.95 | SP=0.991; S=0.895 | 4258 (42.6) | 4211 (98.9) | 47 (1.1) | 492 (10.5) |
| ≥0.96 | SP=0.995; S=0.855 | 4049 (40.5) | 4022 (99.3) | 27 (0.7) | 681 (14.5) |
| ≥0.97 | SP=0.998; S=0.750 | 3540 (35.4) | 3527 (99.6) | 13 (0.4) | 1176 (25.0) |
| ≥0.98 | SP=0.998; S=0.603 | 2844 (28.4) | 2835 (99.7) | 9 (0.3) | 1868 (39.7) |

© 2025 Alves F et al. *JAMA Network Open*.

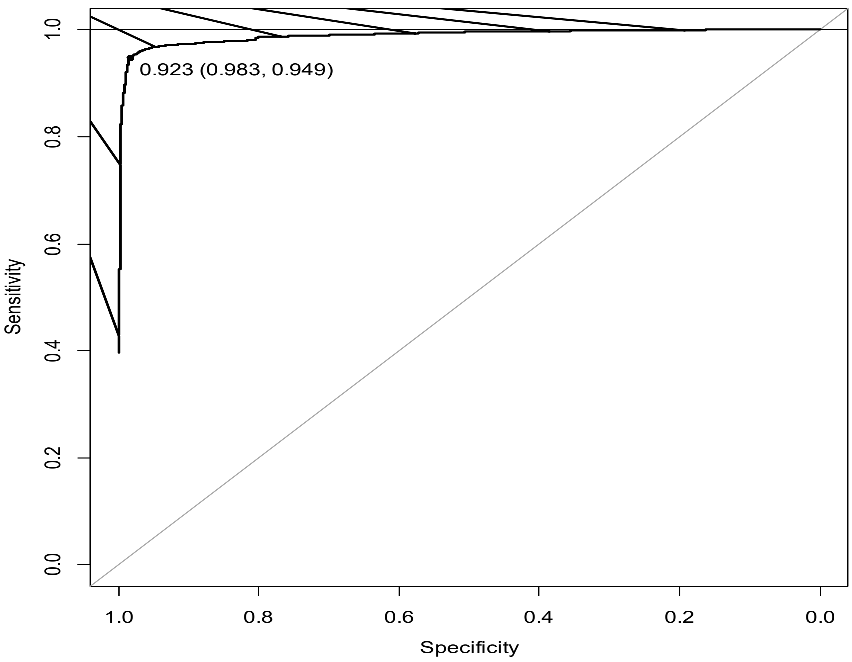


**eFigure 1.** ROC Curve of the linkage between mortality and CadUnico from 2001 to 2015.

## 3. Flowchart of the selected population

Our study population was derived from a cohort of 100 million Brazilians, totaling 131,701,455 individuals distributed among 46,328,173 families during the period from 2001 to 2018. We identified and excluded 237,208 inconsistent records according to the criteria presented in eFigure 2.

© 2025 Alves F et al. *JAMA Network Open*.

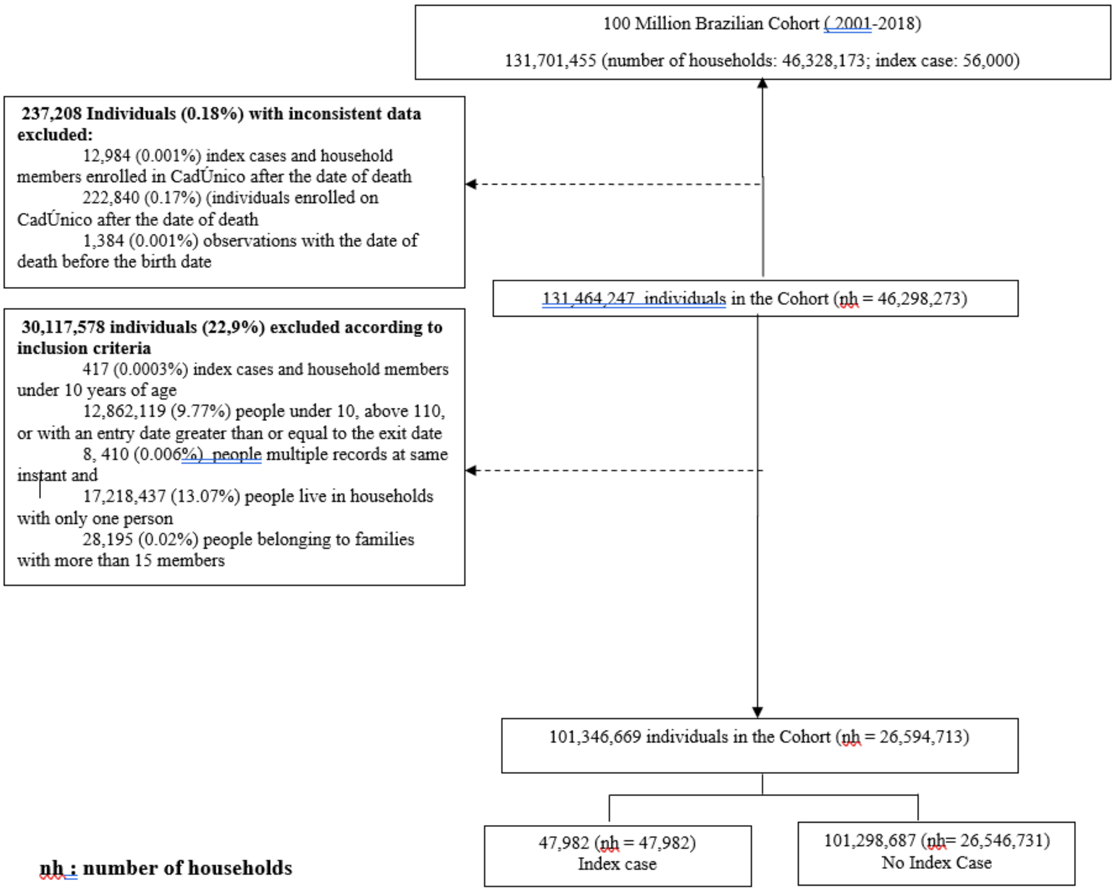


**eFigure 2.** Flowchart of the selected population

Additionally, we applied the following exclusion criteria, resulting in the exclusion of 30,117,578 individuals:

1- **Exclusion of individuals below 10 Years Old and above 110 Years Old:** Since suicides are rare in age groups below 10 years old, we excluded individuals younger than 10 years old at the time of registration or those who would reach 10 years old during our follow-up period (January 1, 2001, to December 31, 2018). If these individuals were index cases, we removed both the index case and their household members. We also excluded individuals aged above 110 years at registration, as this likely led to missing or unlinked death certificates. Additionally, we excluded individuals with an entry date greater than or equal to the exit date.

- For unexposed individuals, the entry date was classified as the date of registration in the cohort or the day they turned 10 years old. The exit date was considered as the date of the suicide index case (since individuals become exposed after that ), the end of follow-up (December 31, 2018), or the date of the individual's death for other causes?.

- For exposed individuals, the entry date was classified as the date of the suicide index case occurrence . The exit date was considered as the end of follow-up (December 31, 2018), or the date of the individual's death.

- These criteria resulted in the exclusion of 12,862,119 individuals (9.77%), along with 417(0.0003%) index cases under 10 years of age and their household members.

© 2025 Alves F et al. *JAMA Network Open*.

2- **Exclusion of dates recorded at the same instant:** Since the follow-up period for each individual must be equal to or greater than 1, we excluded 8,410 (0.0006%) individuals with same records for critical dates (i.e., the date of entry in the cohort being the same as the date of death, or the entry date being equal to the exit date).

3- **Exclusion of Single-Member Households:** Individuals in households with only one member were excluded due to the absence of other individuals who could be at risk for the event. This may be because they were initially registered in the cadastro único as single-person households or they lived with other relatives at the time of enrollment; however, those household members were later excluded during the data cleaning process, based on the study's inclusion and exclusion criteria (n= 17,218,437, 13.07%).

4- **Exclusion Based on Household Size Outliers:** Given the presence of outliers in the number of household members, which may be due to registration errors during the linkage process, we excluded these outliers according to the 99.9th percentile criterion. Consequently, we excluded 28,195(0.02%) individuals from households with more than 15 members (eTable 2).

eTable 2. Summary Measures of the Variable "Number of People in the household"

| Min | Mean | SD | Máx | Percent 99.9 |
|---|---|---|---|---|
| 2 | 4.34 | 1.96 | 61 | 14 |

eTable 3. Descriptive analysis by index case

| Characteristic | Overall, N = 101,346,669 | Index case, N = 47,982 |
|---|---|---|
| Sex | | |
| Male | 47,735,812 (47%) | 36,712 (77%) |
| Female | 53,610,857 (53%) | 11,270 (23%) |
| Age cohort | | |
| 10-24 | 58,916,752 (58%) | 21,266 (44%) |
| 25-59 | 37,328,899 (37%) | 24,705 (51%) |
| 60-110 | 5,101,018 (5.0%) | 2,011 (4.2%) |
| Race/color | | |
| White | 31,126,607 (33%) | 17,122 (39%) |
| Black | 7,047,954 (7.5%) | 3,267 (7.5%) |
| Asian descendants | 373,208 (0.4%) | 140 (0.3%) |
| Brown | 55,453,403 (59%) | 22,169 (51%) |
| Indigenous | 542,080 (0.6%) | 718 (1.7%) |
| Unknown | 6,803,417 | 4,566 |
| Region | | |
| Northeast | 40,127,326 (40%) | 17,218 (36%) |
| North | 10,481,961 (10%) | 4,100 (8.6%) |
| Southeast | 32,368,079 (32%) | 13,485 (28%) |
| South | 11,553,359 (11%) | 9,581 (20%) |
| Central-West | 6,722,316 (6.6%) | 3,542 (7.4%) |
| Unknown | 93,628 | 56 |
| Location residence | | |
| Urban | 73,257,959 (74%) | 32,981 (71%) |
| Rural | 25,379,148 (26%) | 13,520 (29%) |
| Unknown | 2,709,562 | 1,481 |

© 2025 Alves F et al. *JAMA Network Open*.

| | | |
|---|---|---|
| Unemployed | | |
| Yes | 96,472,083 (95%) | 47,191 (98%) |
| No | 4,874,586 (4.8%) | 791 (1.6%) |
| Construction materials | | |
| Uninformed | 3,634,504 (3.6%) | 1,763 (3.7%) |
| Bricks/cement | 72,766,732 (72%) | 31,856 (66%) |
| Wood, Vegetal materials, and other | 24,945,433 (25%) | 14,363 (30%) |
| Sanitation | | |
| Uninformed | 4,305,950 (4.2%) | 1,952 (4.1%) |
| Public network | 42,451,065 (42%) | 17,853 (37%) |
| Septic tank | 14,959,746 (15%) | 7,881 (16%) |
| Homemade septic tank | 24,721,116 (24%) | 12,529 (26%) |
| Ditch or other | 14,908,792 (15%) | 7,767 (16%) |
| Water supply | | |
| Uninformed | 3,634,103 (3.6%) | 1,762 (3.7%) |
| Public network (running water) | 68,271,151 (67%) | 31,315 (65%) |
| Well, natural sources or other | 29,441,415 (29%) | 14,905 (31%) |
| Waste | | |
| Uninformed | 3,634,543 (3.6%) | 1,762 (3.7%) |
| Public collection system | 71,238,402 (70%) | 31,969 (67%) |
| Burned, buried, outdoor disposal or other | 26,473,724 (26%) | 14,251 (30%) |

## 4. Statistical modeling

In our study we considered a time-dependent exposure variable in a Cox regression model, this means that we are dealing with a scenario in which exposure to a risk factor changes over time for each individual, implying adjustment of the model to account for not only independent covariates but also exposure varying over time. Some studies using data in this context, the usual Cox proportional hazards model could not be applied given the violation of the proportional hazards assumption. One approach for using time-varying covariate data is to extend the Cox proportional hazard model to allow time-varying covariates [9,10].

### 4.1 Time varying Cox regression

To estimate the effect using the time-varying Cox regression model, individuals were observed according to the study period as exposed, if there was a suicide in the same household, and unexposed, otherwise, until a recurrent suicide occurred in the same household nucleus or not until end of study [11,12]. We estimated the effect using multiple adjusted Cox regression and with robust standard errors.

The equation for a Cox regression model with a time-dependent exposure variable may be expressed as:

$$h(t|\boldsymbol{X}, Z(t)) = h_0(t) \,.\, \exp[\beta\boldsymbol{X} + \gamma Z(t)],$$

where,

- $h(t|\boldsymbol{X}, Z(t))$, represents the instantaneous risk of failure at time t given the independent covariates $\boldsymbol{X}$ and the time-varying exposure $Z(t)$;
- $h_0(t)$ represents the base hazard function;
- $\beta$ are the coefficients associated with the independent covariates;

- $\gamma$ are the coefficients associated with time-varying exposure.

$Z(t)$ is the exposure variable that changes over time for each individual. This can be modeled in different ways depending on the nature of the time-varying exposure. The construction of the variable exposure, and the Follow-up can be observed in eFigure 3.

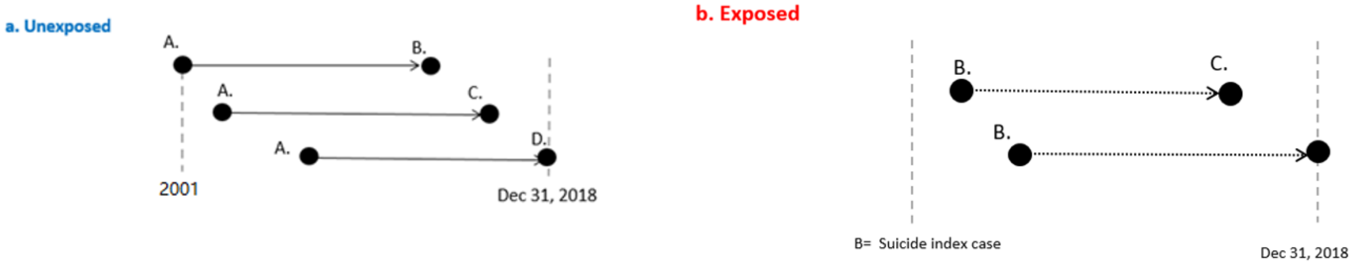


(A)Follow-up began: Cohort entry date or the date the individual turns 10 years old

Follow-up end when:

(B) Suicide index case
(C) Death by suicide or any other cause
(D) Or Dec 31, 2018 (Cohort end date).
*whichever came first

(B) Follow-up began: Suicide index case

Follow-up end when:

(C) Death by any cause;
(D) Dec 31, 2018 (Cohort end date).
*whichever came first

**eFigure 3.** Follow-up

### 4.2 Model check

Checking the proportional hazards assumption after fitting a Cox regression model is the same as identifying time-varying coefficients. In this scenario, we can use the Schoenfeld test, which assesses whether the model's covariates have proportional effects over time. The assumption is that the logarithm of the relative risks (log-hazard ratios) of the independent variables are constant over time (eFigure 4).

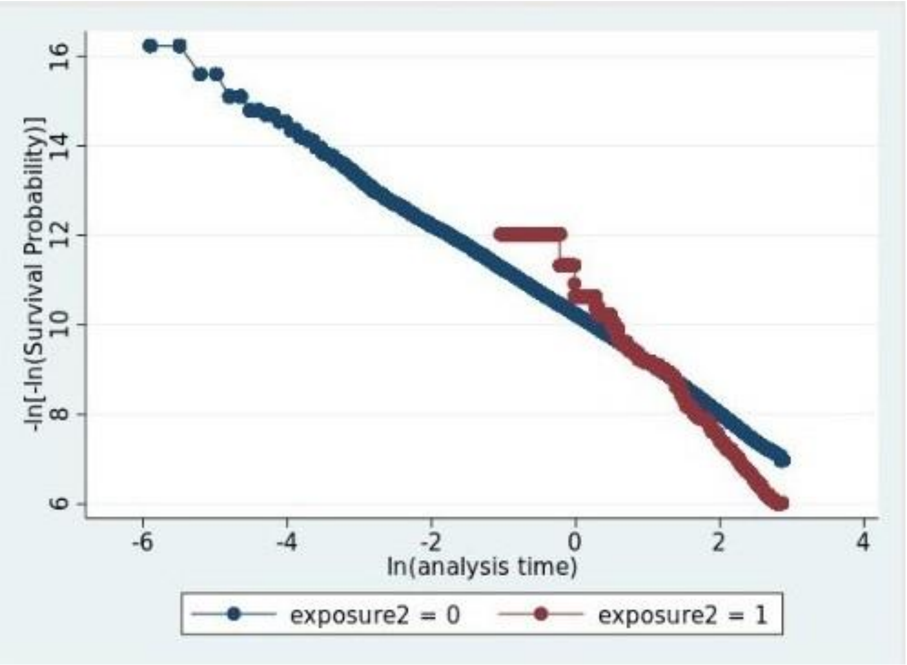


**eFigure 4.** Log-log survival probability

© 2025 Alves F et al. *JAMA Network Open*.

Alternatively, we can observe the plot −ln{−ln(survival)} curves for each category of a nominal or ordinal covariate versus ln(analysis time). These are often referred to as "log–log" plots. Non-parallel curves mean deviations from the proportional hazards assumption. Extreme situations of assumption violation occur when curves intersect. In Figure B we observe this graph in relation to the exposure variable and there is an indication of violation of the proportional risks assumption. In general, an overall significant associated test (p-value = 0.000) is an indication that the time-varying Cox model can be considered (eTable 4).

eTable 4. Test of proportional-hazards assumption

| | Chi2 | df | P-value |
|---|---|---|---|
| Global test | 1431.57 | 37 | 0.0000 |

**4.3 Variable selection for the Cox model using stepwise by characteristics of the index suicide case and sociodemographic characteristics of the survival members among individuals who have experienced a previous suicide within the same household**

Variables for the Cox model were selected using the stepwise method in Stata version [10], with p-value criteria of 0.05 for inclusion (pe) and 0.10 for exclusion (pr). Initially, all variables were included in the selection process and several models were adjusted. We present below the results of two variable selection processes for the outcomes of suicide and all-cause mortality, in which the selected models were those that included the variable living conditions, respectively.

```
. do "/data/tmp/SD2345004.000000"

. stepwise, pr(0.05): stcox Sex_index age_sim_group_index_ref3 Sex_ref_F ///
> age_indiv_obito_anos_group condicao_domic_cat region ///
> race_color location_residence unemployed
                    begin with full model
p = 0.3994 >= 0.0500  removing unemployed

Cox regression -- Breslow method for ties

No. of subjects =      197,353                 Number of obs    =    197,353
No. of failures =       43,177
Time at risk    =  1153749.202
                                               LR chi2(8)       =   18623.29
Log likelihood  =   -468121.24                 Prob > chi2      =     0.0000

------------------------------------------------------------------------------------------
                        _t | Haz. Ratio   Std. Err.      z    P>|z|     [95% Conf. Interval]
---------------------------+--------------------------------------------------------------
                 Sex_index |   1.230772   .0142387    17.95   0.000     1.203179    1.258999
  age_sim_group_index_ref3 |   .9073595   .0070716   -12.47   0.000     .8936047    .9213261
                 Sex_ref_F |   3.060994   .0354062    96.72   0.000      2.99238    3.131182
age_indiv_obito_anos_group |   1.023611   .0003385    70.58   0.000     1.022948    1.024274
        condicao_domic_cat |   .7184131   .0043233   -54.96   0.000     .7099894    .7269367
                    region |    1.05088   .0040027    13.03   0.000     1.043064    1.058755
                race_color |   1.017471   .0035838     4.92   0.000     1.010471    1.024519
        location_residence |    .880967   .0095305   -11.72   0.000     .8624843    .8998458
------------------------------------------------------------------------------------------

.
end of do-file
```

```
. do "/data/tmp/SD2345004.000000"

. stepwise, pr(0.05): stcox Sex_index age_sim_group_index_ref3 Sex_ref_F ///
> age_indiv_obito_anos_group condicao_domic_cat region ///
> race_color location_residence unemployed
                      begin with full model
p = 0.6551 >= 0.0500  removing unemployed

Cox regression -- Breslow method for ties

No. of subjects =      197,353                Number of obs    =    197,353
No. of failures =        3,618
Time at risk    =  1153749.202
                                              LR chi2(8)       =    3647.94
Log likelihood  =   -40058.511                Prob > chi2      =     0.0000

---------------------------------------------------------------------------------------------
                         _t | Haz. Ratio   Std. Err.      z    P>|z|     [95% Conf. Interval]
----------------------------+----------------------------------------------------------------
                  Sex_index |    1.24402   .0488235     5.56   0.000     1.151915    1.343488
   age_sim_group_index_ref3 |   .7723645   .0187911   -10.62   0.000     .7363989    .8100867
                  Sex_ref_F |   1.185353   .0404583     4.98   0.000      1.10865    1.267362
 age_indiv_obito_anos_group |   1.060196   .0009722    63.74   0.000     1.058292    1.062103
         condicao_domic_cat |   .9317402   .0205678    -3.20   0.001     .8922877    .9729372
                     region |   1.037646   .0136769     2.80   0.005     1.011183    1.064802
                 race_color |   1.049685   .0128657     3.96   0.000      1.02477    1.075207
         location_residence |   .7495873   .0288763    -7.48   0.000     .6950746    .8083752
---------------------------------------------------------------------------------------------

.
end of do-file
```

### 4.4 Suicide rates by characteristics of the index suicide case among individuals who have experienced a previous suicide within the same household

Upon calculating all-cause mortality and subsequent suicide rates in the same household based on the characteristics of the index suicide case, we first observed that all-cause mortality rates were highest among household members of index cases aged 25–59 (659.89 per 100,000 person-years, 95% CI = 594.43–732.55), followed by those aged 10–24 (452.34 per 100,000 person-years, 95% CI = 429.15–476.78), and lowest among those aged 60 and older (381.18 per 100,000 person-years, 95% CI = 365.82–397.19). When stratified by the sex of the index case, household members of male index cases experienced higher all-cause mortality (462.19 per 100,000 person-years, 95% CI = 434.18–492.00) compared to those of female index cases (407.37 per 100,000 person-years, 95% CI = 393.11–422.15). Regarding suicide rates, we identified higher rates among household members of young index cases aged 25–59 (33.26 per 100,000 person-years, 95% CI = 27.40–40.39), compared to those of index cases aged 60 and older (19.65 per 100,000 person-years, 95% CI = 16.39–23.50) or aged 10–24 years (24.37 per 100,000 person-years, 95% CI = 14.10–41.97). In terms of the index case's sex, higher suicide rates were observed among household members of female index cases (32.44 per 100,000 person-years, 95% CI = 25.62–41.08), compared to male index cases (21.95 per 100,000 person-years, 95% CI = 18.82–25.58) (eTable 5).

eTable 5. All-cause mortality and suicide rates by characteristics of the index suicide case among individuals who have experienced a previous suicide within the same household, 2001-2018

| Characteristics | All-cause mortality | Person/Years | Rate | 95% CI[1] | Suicide | Person/Years | Rate | 95% CI[1] |
|---|---|---|---|---|---|---|---|---|
| **Overall** | 4009 | 955488.8 | 419.57 | 406.79 432.77 | 232 | 955400.54 | 24.28 | 21.35 27.62 |
| **Characteristics of the index case** | | | | | | | | |

© 2025 Alves F et al. *JAMA Network Open*.

| | | | | | | | | |
|---|---|---|---|---|---|---|---|---|
| **Sex of the Index Case** | | | | | | | | |
| Female | 3026 | 742805.14 | 407.37 | 393.11 422.15 | 69 | 212683.66 | 32.44 | 25.62 41.08 |
| Male | 983 | 212683.66 | 462.19 | 434.18 492.00 | 163 | 742716.87 | 21.95 | 18.82 25.59 |
| **Mortality Age of the Index Case** | | | | | | | | |
| 10-24 | 1387 | 306626.87 | 452.34 | 429.15 476.78 | 13 | 53342.196 | 24.37 | 14.15 41.97 |
| 25-59 | 352 | 53342.196 | 659.89 | 594.43 732.55 | 102 | 306626.87 | 33.26 | 27.40 40.39 |
| 60 years old or older | 2270 | 595519.74 | 381.18 | 365.82 397.19 | 117 | 595431.47 | 19.65 | 16.39 23.55 |
| **Individual characteristics** | | | | | | | | |
| **Sex of the individual** | | | | | | | | |
| Female | 1761 | 516909.68 | 340.68 | 325.13 356.97 | 174 | 438552.58 | 39.68 | 34.20 46.03 |
| Male | 2248 | 438579.12 | 512.56 | 491.81 534.20 | 58 | 516847.96 | 11.22 | 8.68 14.52 |
| **Mortality age of the individual** | | | | | | | | |
| 10-24 | 790 | 552661.54 | 142.94 | 133.32 153.27 | 115 | 552587.66 | 20.81 | 17.33 24.98 |
| 25-59 | 1789 | 357332.56 | 500.65 | 477.98 524.40 | 108 | 357322.33 | 30.22 | 25.03 36.50 |
| 60 years old or older | 1430 | 45494.69 | 3143.22 | 2984.46 3310.43 | 9 | 45490.539 | 19.78 | 10.29 38.02 |
| **Household characteristics** | | | | | | | | |
| **Living conditions** | | | | | | | | |
| 0 | 1249 | 272875.44 | 457.72 | 433.02 483.82 | 69 | 151028.23 | 45.69 | 36.08 57.84 |
| 1 | 1003 | 213723.95 | 469.30 | 441.13 499.26 | 34 | 172790.44 | 19.68 | 14.06 27.54 |
| 2 | 641 | 146067.72 | 438.84 | 406.15 474.16 | 44 | 154297.51 | 28.52 | 21.22 38.32 |
| 3 | 521 | 145849.27 | 357.22 | 327.82 389.25 | 34 | 218535.41 | 15.56 | 11.12 21.77 |
| 4 | 595 | 176972.41 | 336.21 | 310.25 364.34 | 51 | 258748.96 | 19.71 | 14.98 25.93 |
| **Region** | | | | | | | | |
| Northeast | 1524 | 367126.38 | 415.12 | 394.79 436.49 | 65 | 367099.5 | 17.71 | 13.88 22.58 |
| North | 303 | 85837.478 | 352.99 | 315.40 395.06 | 23 | 85837.478 | 26.79 | 17.80 40.32 |
| Southeast | 1088 | 252932.4 | 430.15 | 405.34 456.49 | 45 | 252932.4 | 17.79 | 13.28 23.83 |
| South | 822 | 181792.61 | 452.16 | 422.28 484.15 | 66 | 181767.71 | 36.31 | 28.53 46.22 |
| Central-West | 267 | 66556.82 | 401.16 | 355.82 452.28 | 33 | 66520.335 | 49.61 | 35.27 69.78 |

1The 95% CI estimates take into account the method described by Breslow and Day to calculate the standard error and assume that the numbers of events in each age group follow a Poisson Distribution.

© 2025 Alves F et al. *JAMA Network Open*.

### 4.5 Statistical Model and Interaction Interpretation between Sex of the index case and Age of the index case

A multivariable Cox regression was used to examine factors associated with subsequent suicide or all-cause mortality. To examine differential effects by sex and age, we included an interaction term between the sex and age group of the index case of the surviving household members. The model is specified as follows:

$h\_i(t) = h\_0(t) * \exp(\beta_1 * Sex\_index\_i + \beta_2 * AgeGroup\_index\_i + \beta_3 * Sex\_index\_i \times AgeGroup\_index\_i + X\_i * \beta)$

Where:
- h_i(t) is the hazard function for individual i at time t,
- h_0(t) is the baseline hazard function,
- Sex_index_i is a binary indicator (0 = Female, 1 = Male),
- AgeGroup_index_i is a categorical variable (10–24, 25–59, 60+),
- Sex_index_i × AgeGroup_index_i is the interaction term,
- X_i is a vector of covariates (e.g., race/color, living conditions, region),
- β is the vector of corresponding coefficients.

**Interpretation of the Interaction Effect**

The interaction term $\beta_3$ shows how the effect of sex on the hazard varies across different age categories. In this model, the interaction between sex and age modifies the hazard for all-cause mortality and suicide outcomes. This suggests that the relationship between sex and the hazard of the outcome is not constant across all age groups, but rather changes as age varies.

From the results in the table, we observe the following:

1. **Female Index Case in the Age Group 10-24:**
    - The hazard ratio for female index cases in this age group is 0.72 (adjusted), which indicates that, compared to male index cases aged 60+ (the reference group), females aged 10-24 have a significantly lower risk of all-cause mortality. This suggests that younger females experience a lower risk of mortality relative to older males.
    - For suicide outcomes, the interaction effect suggests a similar pattern, though the confidence intervals are wider, indicating some uncertainty in this estimate (adjusted HR = 0.70).
2. **Female Index Case in the Age Group 25-59:**
    - The adjusted hazard ratio for females in this age group is 0.94 (all-cause mortality), indicating a slightly reduced risk compared to older male index cases, though this effect is not as pronounced as in the younger group.
    - For suicide outcomes, the hazard ratio for females aged 25-59 is 1.88 (unadjusted), which suggests a much higher risk relative to the male reference group, although the confidence intervals are wide (1.78-6.16), indicating a degree of uncertainty about this estimate.

**Summary of Interpretation:**

- Effect of Sex and Age on Mortality and Suicide Outcomes:
    - The interaction between sex and age suggests that younger females (aged 10-24) have a lower risk of all-cause mortality compared to older males, while older females (25-59) show a higher risk of suicide outcomes compared to older males. This highlights the importance of considering both age and sex when assessing the risk of these outcomes.
- Examples:
    - Example 1: A female index case aged 10-24 years old has a lower risk of all-cause mortality (adjusted HR = 0.72) compared to a male index case aged 60+.

© 2025 Alves F et al. *JAMA Network Open*.

- Example 2: A **female index case** aged 25-59 years old is at **higher risk for suicide** (adjusted HR = 1.88) compared to a male index case aged 60+, although there is a high degree of uncertainty in the estimate.

These results underscore the importance of considering both sex and age as interacting factors when analyzing mortality and suicide risks. The interaction suggests that the effects of sex are not uniform across all age groups, and different age categories may require distinct interventions or analyses.

**4.6 Interaction Test Between Time Periods**

To evaluate whether the associations between each covariate and the risk of suicide differ between time periods, we fit Cox proportional hazards models that included a binary indicator of follow-up period (fup_time_suicide) and interaction terms between this indicator and each covariate. For example, we considered the periods ≤2 years and ≥3 years.

**Model Specification**

Let $T_i$ be the survival time for individual $i$ and $h_0(t)$ the baseline hazard. For each covariate vector $X_i$, we defined a fixed indicator of follow-up duration:

$$D_i = 0 \text{ if follow-up } \leq 2 \text{ years;}$$

$$D_i = 1 \text{ if follow-up } \geq 3 \text{ years.}$$

The model was:

$$h_i(t) = h_0(t) \times \exp\{ \beta^t X_i + \gamma D_i + \theta^t (X_i \times D_i) \}.$$

Here, $\theta$ captures the difference in log–hazard ratios between the ≥3-year and ≤2-year groups. Because $D_i$ was defined once at baseline and does not vary within the individual, this is a conventional Cox model (not a model with time-dependent covariates).

**Estimation and Testing**

Models were fit in Stata [10] using the stcox command (Breslow method for ties). For each covariate, a Wald test of $H_0$: $\theta = 0$ was performed with the testparm command to jointly assess all interaction coefficients. The resulting *P-value* is reported in figures/tables as *P_diff*, representing the significance of the difference in association between the early and late follow-up periods.

Interpretation.
A *P_diff* < *.05* indicates evidence that the hazard ratio for the covariate of interest differs between the ≤2-year and ≥3-year follow-up groups.

**Illustrative Stata Syntax**

```
* Cox model with period × characteristic interaction

stcox i.Sex_ref_F##i.fup_time_suicide ///
i.age_indiv_obito_anos_group##i.fup_time_suicide ///
i.Sex_index##i.fup_time_suicide ///
i.age_sim_group_index_ref3##i.fup_time_suicide ///
i.region##i.fup_time_suicide ///
i.living_conditions##i.fup_time_suicide

* Joint Wald test of k-th predictor interactions

testparm i.Sex_index#i.fup_time_suicide   // example of interaction test
```

For further details on the interaction test, see [14, 15].

© 2025 Alves F et al. *JAMA Network Open*.

## 5. Sensitivity analyses

In our main analysis, follow-up was defined as follows: unexposed individuals were followed from their cohort entry date until the earliest of (i) death from any cause (including suicide), (ii) end of follow-up (December 31, 2018), or (iii) the occurrence of a suicide index case within the same household. Exposed individuals were followed from the date of the suicide index case until the earliest of (i) death from any cause (including suicide) or (ii) end of follow-up (December 31, 2018).

Given the different follow-up definitions for exposed and unexposed groups, we conducted additional specifications to test the robustness of our findings. We re-estimated the models under three alternative approaches: (1) applying a doubly robust model by further adjusting the main analysis for individual follow-up time, (2) assigning both exposed and unexposed individuals the start of follow-up at cohort entry, and (3) simulating a model in which all cohort members had equal follow-up duration. All approaches yielded results consistent with those of the main analysis, supporting the robustness of our findings.

### 5.1 Time-Dependent Exposure and Explicit Modeling of Follow-up Duration

This section presents a sensitivity analysis in which the exposure to a household suicide index was treated as a time-dependent variable and the duration of the follow-up was explicitly modeled. The modeling strategy treats "time of exposure" as the time since the household index event. Coding was implemented as follows: before exposure, time_expo = 0 for all intervals; after exposure, time_expo = t – t_expo (in years) [15, 16, 17].

The Cox model included two key time-varying terms: (1) an indicator of being exposed (EXPO), which switches from 0 to 1 at the index event and captures the immediate jump in risk, and (2) the continuous variable time_expo, which grows only after the cut point and captures the gradient of risk with exposure duration.

This parameterization aligns the risk sets to an appropriate zero time for each group and prevents immortal time bias. It ensures that no person-time is classified as "exposed" before the suicide event in the household. It estimates separately the immediate effect of becoming exposed ($\beta_1$) and the effect of the duration of exposure ($\beta_2$), as

$$h(t) = h_0(t)\, exp\{ \beta_1 \cdot EXPO(t) + \beta_2 \cdot time\_expo(t) + \beta' X \},$$

where $\beta_1$ represents the instantaneous change in hazard at exposure and $\beta_2$ represents the multiplicative change in hazard per additional year of exposure; for non-exposed or pre-exposure periods, time_expo = 0.

After model adjustment, for suicide mortality, becoming exposed to an index suicide in the household was associated with a 3.5-fold increase in the risk of suicide at the time of exposure (HR = 3.53; 95% CI, 2.97–4.21) and for all-cause mortality, exposure was associated with an immediate 8% increase in risk (HR = 1.08; 95% CI, 1.04–1.13) (eTable 6). These findings confirm that the main associations remain when explicitly considering duration of exposure.

eTable 6. Hazard Ratios for Suicide and All-Cause Mortality Including Time-Dependent Exposure and Duration of Follow-up

| Outcome | Exposure indicator (EXPO)a HR (95% CI) | P value | Subjects / Events |
|---|---|---|---|
| **Suicide** | 3.53 (2.97–4.21) | <0.001 | 93,769,418 / 43,177 |

© 2025 Alves F et al. *JAMA Network Open*.

| | | | |
|---|---|---|---|
| **All-cause mortality** | 1.08 (1.04–1.13) | <0.001 | 93,769,418 / 3,118,303 |

a EXPO = binary indicator switching from 0 to 1 at the date of the household index suicide, representing the immediate change in hazard.

## 5.2 Common Definition of Follow-up Entry for Exposed and Unexposed

To assess the potential influence of differential follow-up time, we performed a sensitivity analysis in which the start date for all participants—both exposed and unexposed—was defined as the date of cohort entry. Survival time was therefore calculated from the common cohort entry date until the individual exit date. Cox proportional hazards models were fitted with the same covariate adjustment as the main analysis.

Exposure to a suicide index case remained strongly associated with suicide risk (hazard ratio [HR] = 4.65; 95% CI, 4.06–5.33) and all-cause mortality (HR = 1.32; 95% CI, 1.27–1.36) (eTable 7). These estimates were consistent with the primary results, indicating that the observed associations were not driven by differences in follow-up time.

eTable 7. Hazard Ratios for Suicide and All-Cause Mortality Using a Common Definition of Follow-up Entry for Exposed and Unexposed

| Outcome | HR (95% CI) | P value | Subjects / Events |
|---|---|---|---|
| **Suicide** | 4.65 (4.06–5.33) | <0.001 | 93,769,418/ 43,177 |
| **All-cause mortality** | 1.32 (1.27–1.36) | <0.001 | 93,769,418/ 3,118,303 |

### 5.2.1 Distribution of Follow-up Time

Comparing the two definitions of follow-up initiation—dt_entry_10 (the follow-up used in the main model, with cohort entry for unexposed and date of index case for exposed) versus dt_entry_cohort_10 (cohort entry for both exposed and unexposed)—produced nearly identical follow-up distributions. In both specifications, unexposed participants had a median follow-up of approximately 11.3 years, whereas exposed participants had a median of about 5 years. Mean follow-up was likewise consistent (≈9.7 years for unexposed and ≈5.7 years for exposed) (eTable 6). These findings show that the choice of baseline date did not materially affect time-at-risk estimates.

Because the exposed group necessarily enters follow-up on the date of the index household suicide, we evaluated whether alternative definitions of cohort entry altered the distribution of follow-up time. Using either dt_entry_10 or data_entrada_10 as the time origin yielded nearly identical estimates of median and mean follow-up within exposure strata (eTable 8). These findings indicate that our results are robust to the definition of baseline time and support the use of time-varying exposure in the main Cox models to prevent immortal-time bias and accurately represent differential follow-up [15, 16, 17].

eTable 8. Distribution of follow-up time (years) by definition of baseline date and exposure status

| Baseline definition | Exposure | N (subjects) | Median (p50) | p25–p75 | Mean (SD) |
|---|---|---|---|---|---|
| **dt_entry_10**[1] | 0 (unexposed) | 1.01 × 10^8 | 11.34 | 6.09–12.74 | 9.74 (4.63) |
| | 1 (exposed) | 167,472 | 5.22 | 2.36–8.64 | 5.71 (3.86) |
| **dt_entry_cohort_10**[2] | 0 (unexposed) | 1.01 × 10^8 | 11.34 | 6.09–12.74 | 9.74 (4.63) |

© 2025 Alves F et al. *JAMA Network Open*.

| | 1 (exposed) | 167,472 | 5.21 | 2.12–8.44 | 5.75 (3.92) |
|---|---|---|---|---|---|

1= the variable used in the main model, defined as cohort entry for unexposed and date of index case for exposed
2= cohort entry for both exposed and unexposed

**5.4 Common Follow-up Windows for Exposed and Unexposed**

We performed a sensitivity analysis to evaluate potential follow-up time biases by setting the same follow-up for all individuals. In the multivariable Cox model, exposure to a family index suicide case remained strongly associated with suicide mortality (HR = 3.43; 95% CI, 2.26–5.20). Similarly, family exposure to suicide was positively associated with all-cause mortality (HR = 1.33; 95% CI, 1.18–1.49) (eTable 9). Results were consistent with the primary analysis even after imposing identical follow-up windows for exposed and unexposed groups. This reduces the possibility that differences in follow-up time (for example, more recent entry of exposed individuals) explain the observed associations. The effect sizes—especially for suicide mortality—remained high, reinforcing the robustness of the conclusion that a family member's suicide substantially increases the subsequent risk of both suicide and all-cause mortality.

eTable 9. Hazard Ratios for Suicide and All-Cause Mortality Using Common Follow-up Windows for Exposed and Unexposed

| Outcome | HR (95% CI) | P value | Subjects / Events |
|---|---|---|---|
| **Suicide** | 3.43 (2.26–5.20) | <0.001 | 93,633,187 / 42,990 |
| **All-cause mortality** | 1.33 (1.18–1.49) | <0.001 | 93,633,187 / 3,114,958 |

**5.5 Alternative Follow-up Periods**

The decision to choose periods <=2 vs >=3 as cutoff points was guided both by the literature and by the empirical patterns present in our data. Previous population-based studies of all-cause mortality and suicide have reported that excess suicide risk among surviving relatives is highest in the first 24 months and stabilizes thereafter [18, 19]. For example, [21] demonstrated that excess suicide mortality is particularly high in the first 5 years—and especially in the first year—after an index event. Similarly, population mortality studies reported that excess mortality risk in bereaved parents is concentrated in the first two years post-loss [22]. Moreover, longitudinal studies of bereavement show increased rates of psychiatric outcomes in the initial 1–2 years after loss [18, 20]. These findings motivated our a priori categorization of ≤2 years as the immediate period. Furthermore, in our cohort, visual inspection of the proportions of all-cause death and subsequent suicide after an index family suicide case revealed a marked accumulation of cases through the second year for both outcomes, with a relatively stable proportion after the third year, further corroborating these cutoffs (Figure 2 in the manuscript).

We fitted Cox proportional hazards models including the same covariates as the main analysis, but replaced the binary indicator of follow-up with a categorical variable for these three periods (Immediate (≤1 year), Intermediate (2-4 years), and Distant (≥5 years) and their interactions with each covariate. The resulting forest plot (eFigure 5) shows hazard ratios for all-cause mortality and suicide consistent with the main analysis. P-values for interaction remained >0.05 for nearly all covariates, indicating that the main conclusions are robust to these alternative timescale definitions.

These findings support the a priori choice of ≤2 years and ≥3 years used in the main analysis, while demonstrating that the observed associations remain stable when follow-up is categorized as ≤1 year, 2–4 years, and ≥5 years.

The eFigure 6 presents a visual summary of the key factors significantly associated with increased or decreased risk of (1) all-cause mortality excluding suicide and (2) suicide-specific mortality among household members exposed to a suicide. Results are based on adjusted hazard ratios from

multivariable Cox proportional hazards models. Only factors with statistically significant associations ($p < 0.05$) are displayed.

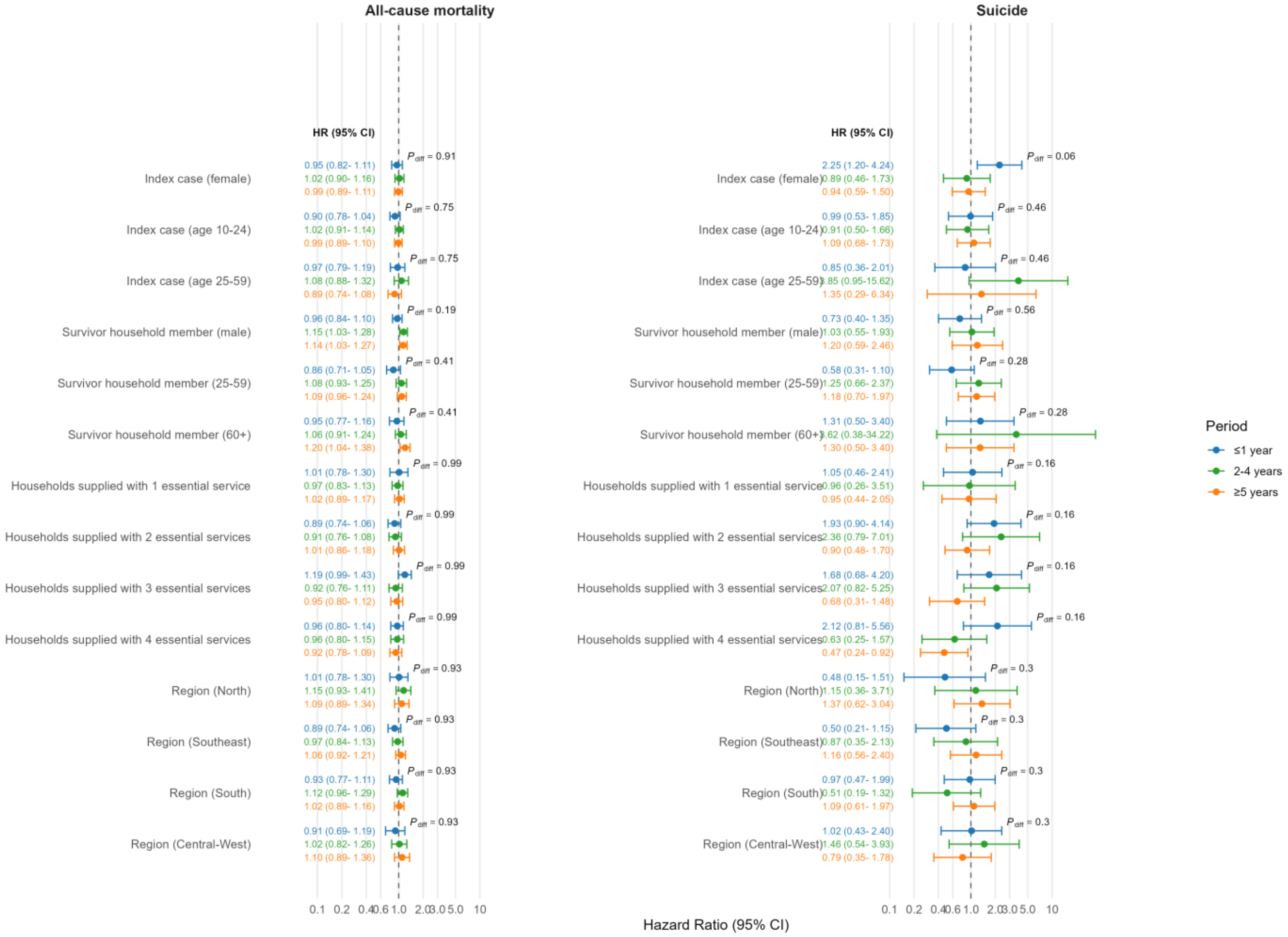


**eFigure 5.** Factors Associated with Immediate (≤1 year), Intermediate (2-4 years), and Distant (≥5 years) Deaths by All-Cause Mortality (Excluding Suicide) and Suicide, 2001–2018. *P_diff represents the test of interaction between the time elapsed since the death of the index case (≤1 year vs. 2-4 years vs. ≥5 years) and the respective characteristic under evaluation.

© 2025 Alves F et al. *JAMA Network Open*.

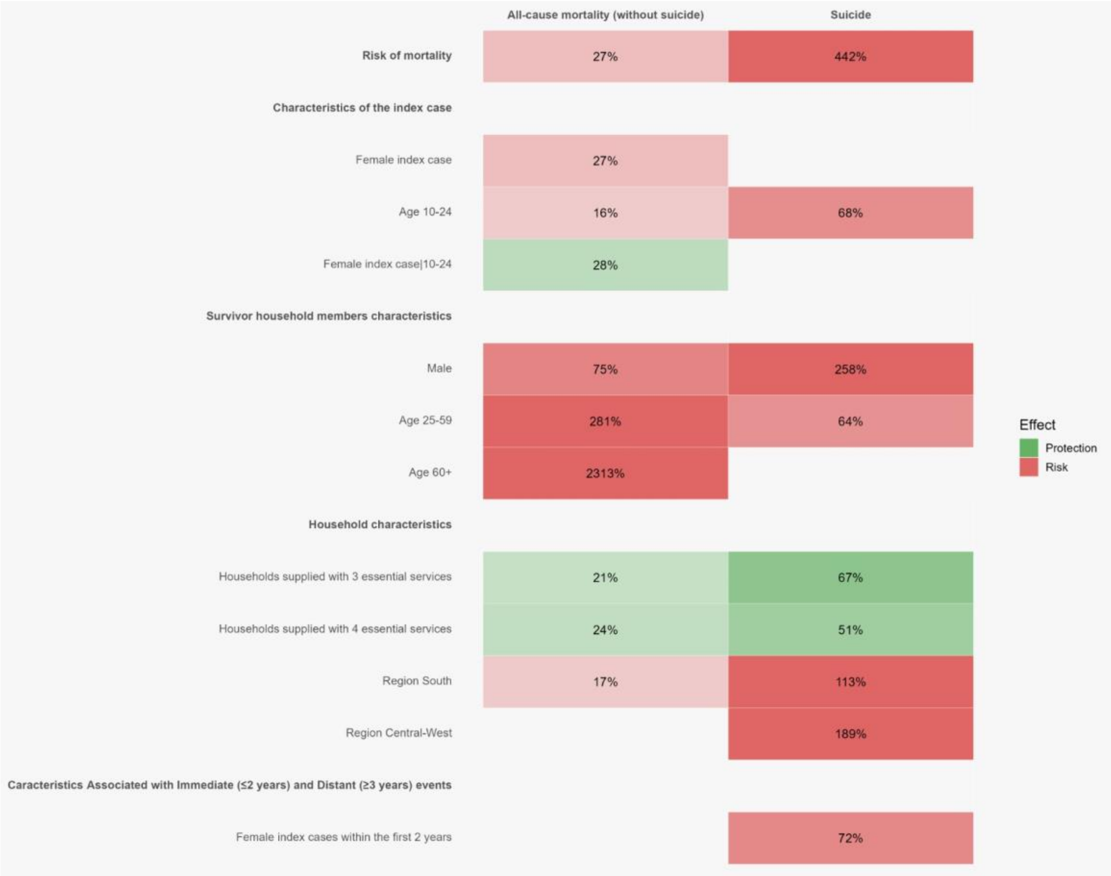


**eFigure 6.** Summary of Statistically Significant Risk and Protective Factors for All-Cause Mortality (Excluding Suicide) and Suicide

## 6. Pathways linking household suicide exposure to subsequent mortality and target groups for postvention interventions

Our cohort study examined the timing and risk factors for all-cause and cause-specific mortality following household exposure to suicide. Such exposure was associated with a 27% increase in all-cause mortality (excluding suicide) and a more than fourfold higher risk of suicide, with a population attributable fraction of 77%. We also identified risk factors for the recurrence of suicide within families. Risks were higher when the index case was younger or female, and when surviving household members were male, aged 25–59 years, or living in households with poor infrastructure. More than half of subsequent suicides occurred within the first two years, underscoring the urgency of targeted early interventions. Based on these findings, eFigure 7 illustrates the pathways linking household suicide exposure to subsequent mortality, as well as the target groups for postvention interventions identified in our study.

© 2025 Alves F et al. *JAMA Network Open*.

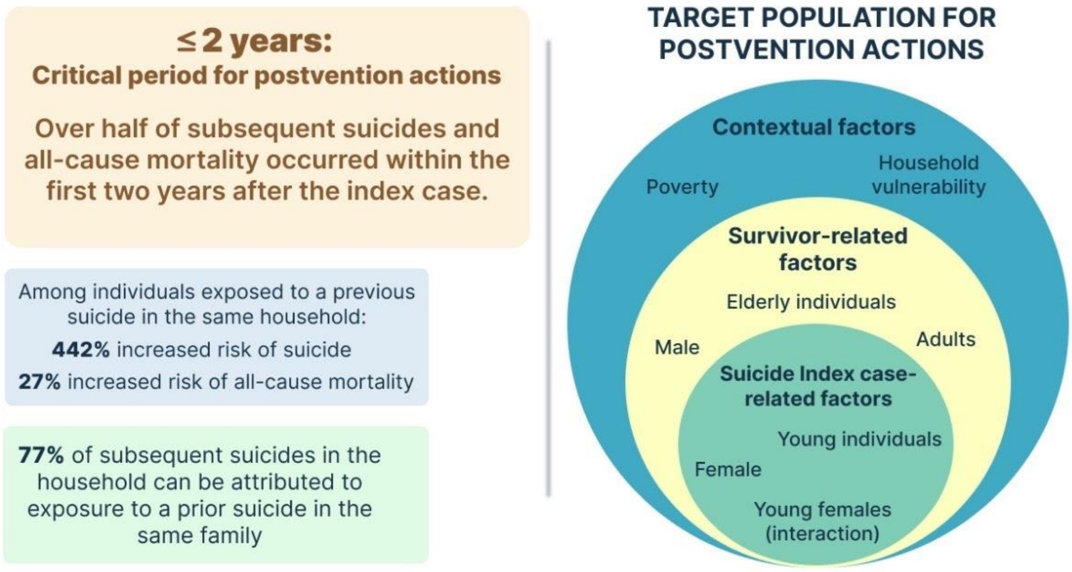


**eFigure 7.** Pathways linking household suicide exposure to subsequent mortality and targets for postvention interventions

## References

1. Sanni Ali M, Ichihara MY, Lopes LC, et al. Administrative data linkage in Brazil: Potentials for health technology assessment. Front Pharmacol 2019;**10**(SEP):1–20.

2. Barreto ML, Ichihara MY, Pescarini JM, et al. Cohort Profile: The 100 Million Brazilian Cohort. Int J Epidemiol 2022; 51: e27–38.

3. DATASUS. Ministério da Saúde [Internet]. [cited 2022 Dec 20]; Available from: https://datasus.saude.gov.br/

4. Pita R, Pinto C, Sena S, et al. On the Accuracy and Scalability of Probabilistic Data Linkage over the Brazilian 114 Million Cohort. IEEE J Biomed Heal Informatics 2018;**22**(2): 346–53.

5. Pinto C, Pita R, Barbosa G, et al. Probabilistic Integration of Large Brazilian Socioeconomic and Clinical

Databases. Proc - IEEE Symp Comput Med Syst 2017;2017–June:515–20.

6. Barreto, Marcos1, Alves, André, Sena, Samila, Fiaccone, Rosemeire, Amorim, Leila, Ichihara, Maria Yuri, and Barreto M. Assessing the accuracy of probabilistic record linkage of social and health databases in the 100 million Brazilian cohort. Int J Popul Data Sci 2017; 2017;**1**(1):276

7. Pita R, Pinto C, Barreto M, et al. Design and evaluation of probabilistic record linkage methods supporting the Brazilian 100-million cohort initiative. Int J Popul Data Sci 2017;**1**(1):23889.

8. Barbosa GCG, Ali MS, Araujo B, et al. CIDACS-RL: a novel indexing search and scoring-based record

linkage system for huge datasets with high accuracy and scalability. BMC Med Inform Decis Mak 2020;

**20**:289.

9. Ahrens, K., Lash, T. L., Louik, C., Mitchell, A. A., & Werler, M. M. Correcting for exposure misclassification using survival analysis with a time-varying exposure. *Annals of epidemiology*, 2012, 22(11), 799-806.

10. StataCorp. (2027). *Stata Statistical Software: Release 15.1.* College Station, TX: StataCorp LLC.

11. Therneau, Terry M. "Extending the Cox model." Proceedings of the first Seattle symposium in biostatistics: survival analysis. New York, NY: Springer US, 1997.

12. Thomas, L., & Reyes, E. M. Tutorial: survival estimation for Cox regression models with time-varying coefficients using SAS and R. Journal of Statistical Software, 2014, 61, 1-23.

13. Zhang, Z., Reinikainen, J., Adeleke, K. A., Pieterse, M. E., & Groothuis-Oudshoorn, C. G. Time-varying covariates and coefficients in Cox regression models. *Annals of translational medicine*, 2018, *6*(7).

14. Cox DR. Regression Models and Life-Tables. J R Stat Soc Series B. 1972;34(2):187-220.

15. Therneau TM, Grambsch PM. Modeling Survival Data: Extending the Cox Model. New York, NY: Springer; 2000.

16. Allison PD. Survival Analysis of Time-Dependent and Time-Varying Covariates. Sociol Methodol. 2010;40(1):211-251.

17. Hernán MA, Sauer BC, Hernández-Díaz S, Platt R, Shrier I. Specifying a target trial prevents

immortal time bias and other self-inflicted injuries in observational analyses. J Clin Epidemiol. 2016;79:70-75.

18. Erlangsen A, et al. Association Between Spousal Suicide and Mental, Physical, and Social Health Outcomes: A Longitudinal Register–Based Study. JAMA Psychiatry. 2017;74(9):933-940.

19. Pitman A, Osborn D, King M, Erlangsen A. Effects of suicide bereavement on mental health and suicide risk. Lancet Psychiatry. 2014 Jun;1(1):86-94.

20. Pham S, Porta G, Biernesser C, Walker Payne M, Iyengar S, Melhem N, Brent DA. The Burden of Bereavement: Early-Onset Depression and Impairment in Youths Bereaved by Sudden Parental Death in a 7-Year Prospective Study. Am J Psychiatry. 2018 Sep 1;175(9):887-896.

21. Probert-Lindström S, Öjehagen A, Ambrus L, Skogman Pavulans K, Berge J. Excess mortality by suicide in high-risk subgroups of suicide attempters: a prospective study of standardised mortality rates in suicide attempters examined at a medical emergency inpatient unit. BMJ Open. 2022 May 27;12(5):e054898.

22. Song J, Mailick MR, Greenberg JS, Floyd FJ. Mortality in parents after the death of a child. Soc Sci Med. 2019 Oct;239:112522.

© 2025 Alves F et al. *JAMA Network Open*.
