# Supplementary material for: Mortality Risk Following a Household Suicide
Source: JAMA Netw Open. 2025 Nov 25;8(11):e2545286. doi: 10.1001/jamanetworkopen.2025.45286 (PMC12648350; doi:10.1001/jamanetworkopen.2025.45286)
Supplement: Supplement 2. — Data Sharing Statement [file jamanetwopen-e2545286-s002.pdf]

Data Sharing Statement

Alves. Mortality Risk Following a Household Suicide. *JAMA Netw Open*. Published November 25, 2025. doi:10.1001/jamanetworkopen.2025.45286

Data
Data available: Yes
Data types: Data dictionary
How to access data: The data dictionary can be obtained by accessing the provided link: https://cidacs.bahia.fiocruz.br/en/platform/cohort-of-100-million-brazilians Please note that all requests will be carefully evaluated, and ethical considerations in research will be taken into account during the evaluation process.
When available: With publication

Supporting Documents
Document types: Statistical/analytic code
How to access documents: Access to the statistical/analytic code can be requested by the link provided https://cidacs.bahia.fiocruz.br/en/platform/cohort-of-100-million-brazilians Please note that all requests will be carefully evaluated, and ethical considerations in research will be taken into account during the evaluation process.
When available: With publication

Additional Information
Who can access the data: Researchers whose proposed use of the data has been approved. Please note that all requests will be carefully evaluated, and ethical considerations in research will be taken into account during the evaluation process.
Types of analyses: For any purpose. Remembering that all requests will be carefully evaluated, and ethical considerations in research will be taken into account during the evaluation process.
Mechanisms of data availability: R: After approval of a proposal, and evaluation of ethical considerations, and with a signed data access agreement
